# Harnessing cosmic carbon: anaerobic microbial responses to fullerenes under early Earth conditions

**DOI:** 10.3389/fmicb.2025.1511842

**Published:** 2025-08-04

**Authors:** Elle Bethune, Andrey Gromov, Eleanor E. B. Campbell, Charles S. Cockell

**Affiliations:** ^1^UK Centre for Astrobiology, School of Physics and Astronomy, University of Edinburgh, Edinburgh, United Kingdom; ^2^Eastchem and School of Chemistry, University of Edinburgh, Edinburgh, United Kingdom

**Keywords:** fullerene, fullerol, C60, C70, anaerobic, microorganism, bacteria, early Earth

## Abstract

Fullerenes of extra-terrestrial origin may have been accessible as carbon sources for anaerobic microorganisms on the early Earth. Very little is known about how anaerobic microorganisms respond to and use fullerenes and their soluble derivatives. We present an investigation into the effects of fullerenes C_60_ and C_70_ and their hydroxylated fullerol derivatives on an environmentally relevant anaerobic community and a microbial isolate. Fullerenes and fullerols irradiated with 254 nm UV radiation for 2 weeks in the absence of oxygen to simulate UV irradiation under anoxia on early Earth were also assessed. The anaerobic community could grow using glucose in the presence of C_60_ up to 500 mg/mL without inhibitory effects on growth. Concentrations of C_70_ of 500 mg/ml were inhibitory. We attribute these results to the different chemical reactivity and photophysical properties of the fullerenes. The experiments suggest the potential for the use of C_60_ as a sole carbon source. Both C_60_ and C_70_ fullerols were inhibitory to growth in the presence of glucose, especially when exposed to light. When we exposed C_60_ fullerol suspensions to 254 nm UV radiation under an anoxic atmosphere, they become significantly more inhibitory to both the community and the isolate, but only if the cultures are grown under ambient light exposure. The anaerobic isolate was unable to grow on C_60_ alone, but after UV radiation exposure, the C_60_ photodegradation products served as a potentially accessible carbon source. Our data show that fullerenes and their derivatives are biologically active and capable of influencing growth in anoxic environments such as those that would have been prevalent on early Earth or in modern-day anoxic soils. Our results show that carbon sources such as these can be both beneficial or deleterious to life depending on their concentrations and environmental processing.

## 1 Introduction

There was likely a significant amount of extra-terrestrial organic carbon present on the early Earth when microbial life first emerged. Investigating the impact of this carbon on primitive microorganisms is crucial for comprehending the origins of early life on Earth, as well as for understanding the potential for life on other young planets.

Carbonaceous chondrites are composed of approximately 3–5% carbon, the majority of which is in the form of insoluble macromolecules (Botta and Bada, 2002; Chyba et al., 1990). An estimated total of 10^20^kg of extra-terrestrial organic material has been accreted by the Earth, at least 10^9^kg of which was deposited during the Late Heavy Bombardment (Chyba et al., 1990; Ehrenfreund et al., 2011). Life is likely to have originated sufficiently early that it would have interacted with this Late Heavy Bombardment reservoir of extra-terrestrial organic material. Although the nature of these interactions is not yet understood, it is possible that the high concentration of organics provided an energy source for the growth of early microorganisms either in fermentative metabolism or as electron donors for other anaerobic metabolic processes (Pasek and Lauretta, 2008; Schönheit et al., 2016).

Fullerenes are a class of compounds characterized by their carbon cage “buckyball” structure which exist in the interstellar medium (Maier and Campbell, 2017) and organic-rich meteorites in amounts ranging from 15 to 28 ppm (Sabbah et al., 2022). Very little is known about how modern microorganisms interact with fullerenes. The limited number of studies that do address this usually do so from an ecological standpoint stemming from the risk fullerenes and related carbon nanomaterials pose to the environment as contaminants (Liu et al., 2023; Zuo et al., 2024; Leroy et al., 2024). Fullerene C_60_, the most stable and therefore most abundant and well-studied fullerene, is widely reported to be bactericidal under aerobic conditions (Chae et al., 2014; Duncan et al., 2008; Fang et al., 2007; Fortner et al., 2005; Hancock et al., 2012; Lyon et al., 2006; Tsao et al., 2002). However, it does not appear to be inhibitory to anaerobic microorganisms in anoxic environments as the presence of C_60_ is not found to significantly alter gas production or community composition in anaerobic wastewater reactors (Nyberg et al., 2008; Zhao et al., 2018).

Although C_60_ appears to be resistant to mineralization in soils over long periods (Avanasi et al., 2014), there is some evidence to suggest it may be susceptible to microbial degradation under certain conditions (Chae et al., 2014). In 2020, it was reported that the aerobic bacterial species *Labrys.* sp. WJW could degrade C_60_ by excreting a siderophore to facilitate a Fenton-like biodegradation reaction to break the fullerene cage structure (Wang et al., 2020). To the best of our knowledge at the time of this paper, there is no report of biodegradation of any fullerene by an anaerobic microorganism. This paucity of investigations could be because most environments where the effects of fullerene contamination are of concern are aerobic, and therefore fewer studies have focused on anaerobic species.

Fullerene C_60_ has very low water solubility in its native form (Avanasi et al., 2014; Lyon et al., 2006; Ruoff et al., 1993). On contact with water, it forms stable colloidal aggregates, known as nC_60_, which possess different chemical properties to bulk C_60_ (Avanasi et al., 2014; Duncan et al., 2008; Lyon et al., 2006). Therefore, it is these nC_60_ aggregates that microorganisms interact with in aqueous media, both in the laboratory and the environment. Furthermore, C_60_ is readily altered by exposure to environmental conditions, therefore it is highly likely that certain factors on the early Earth would have impacted the chemistry of the fullerenes present, and therefore changed their accessibility to, and effects on, early microorganisms.

In addition to fullerenes, another class of molecules of potential interest on early Earth are the fullerols. Fullerol C_60_ is a derivative of fullerene C_60_ characterized by the attachment of many hydroxyl functional groups (Semenov et al., 2016). Fullerol C_60_ is highly photosensitive and soluble in water (Semenov et al., 2016). Although it still tends to form spherical aggregates in aqueous solutions, this is to a lesser extent than native C_60_ (Brant et al., 2007; Hotze et al., 2009). Fullerol C_60_ is much more bioavailable in soils than its fullerene counterpart, as it is readily mineralized to CO_2_, albeit at a much slower rate than highly bioavailable carbon sources such as glucose (Navarro et al., 2015). Additionally, C_60_ fullerol is readily taken up and converted into microbial biomass by a range of microorganisms in soils under conditions where pristine C_60_ is not mineralized in any detectable amount (Berry et al., 2017), indicating this particular fullerene derivative may be much more accessible as a carbon source for microbes.

Yet another set of derivative chemical compounds are produced by irradiation of the aforementioned molecules. Aqueous C_60_ is readily degraded to produce water-soluble intermediates when exposed to solar-wavelength light in the presence of oxygen (Hou et al., 2010; Hou and Jafvert, 2009), and these degradation products are much more readily degraded by soil bacteria than unaltered C_60_ (Berry et al., 2016). On the early Earth, any aqueous C_60_ would have been in a largely anoxic environment and surface ultraviolet (UV) radiation levels would have been much higher with wavelengths down to 200 nm likely reaching the surface of the planet (Cnossen et al., 2007; Cockell, 1998). C_60_ degraded under UV radiation at 254 nm forms products that are significantly less antibacterial to aerobic bacteria than unaltered C_60_ (Lee et al., 2009). Conversely, C_60_ fullerol is highly photosensitive and produces a range of reactive oxygen species such as singlet oxygen and hydroxyl radicals when exposed to UV radiation (Brunet et al., 2009; Chae et al., 2009; Ma et al., 2020), which has been found to induce lipid and protein oxidation in membranes (Kamat et al., 2000), potentially indicating an increase in antimicrobial activity upon exposure to light.

It is thought that fullerene C_60_ in organic solvents still degrades to some extent under UV radiation when no oxygen is present (Taylor et al., 1991). Very little is known about how C_60_ and fullerol C_60_ behave in anoxic aqueous media when exposed to UV radiation. Any degradation products, including reactive oxygen species, formed under these conditions are likely to be most similar to those that would have been produced on the early Earth, just as any antibacterial effects produced by photoactivated fullerol likely represent what may have occurred in early microbial systems.

In addition to the C_60_ fullerenes and fullerols, there are larger structures that could be biologically relevant. Fullerene C_70_ is the second most stable high molecular weight fullerene and is found in amounts of a similar magnitude to C_60_ (Kroto, 1988). There are very few reported studies concerning the effect of C_70_ on microorganisms, either as an inhibitory species or a subject of biodegradation. It is reported that C_70_ derivatives exert antimicrobial activity toward both Gram-positive and Gram-negative bacteria (Huang et al., 2013), likely due to the release of reactive oxygen species from the functionalized C_70_ particles which attack bacterial cell walls (Huang et al., 2014, 2013; Ouyang et al., 2016). However, there is little literature available to support this.

In this study, the effects of native and photodegraded fullerenes C_60_ and C_70_ and their fullerol derivatives on anaerobic microorganisms were examined. An anaerobic community that was previously selected to grow using raw meteorite material as a carbon source by Waajen et al. (2022) was used in these experiments as an analog of early heterotrophic microbial life. An isolate obtained from this community was used in additional experiments to compare the effects of fullerenes on a community with a single related organism. Aqueous C_60_ and C_70_ were irradiated with 254 nm UV radiation under anoxic conditions and the degradation products and their effects on both the anaerobic community and isolate were characterized and compared with the corresponding native fullerenes. We discuss these results in the context of early life on Earth. These results have implications for the modern challenge of understanding the effects of human fabricated carbon nanomaterials in the environment.

## 2 Materials and methods

### 2.1 Fullerene suspensions

Powdered fullerene C_60_ (99.5%+ purity) and C_70_ (98%+ purity) were acquired from IOLITEC GmbH (Germany) and MST (Latvia), respectively. One hundred mg/L aqueous suspensions of fullerene C_60_ were prepared by the following method: powdered fullerene was added to sterile M9 minimal media and placed in a sonicator bath (Elma S 60 H Elmasonic) for 6 h until no large aggregates were visible, and the medium appeared dark gray and cloudy. The sonicator bath temperature was kept below 45°C by adding ice water every 30 min.

Fullerol C_60_ and C_70_ were synthesized and characterized according to a procedure adapted from Kokubo et al. (2011). Firstly, C_60_ (150 mg) was dissolved in 150 mL toluene and then 25 mL of 30% H_2_O_2_ was added slowly, followed by 0.8 mL of 40% aqueous solution of Tetrabutylammonium hydroxide (Bu_4_NOH). The mixture was stirred (750–1,000 rpm) at 60 °C until complete decolourization of the organic layer was observed (10–15 h). The toluene and aqueous layers were then separated. The evaporation of water/H_2_O_2_ (55–60°C; < 20 mbar) was continued until the consistency of the residue resembled soft wax. The residue was diluted with 30 mL of isopropyl alcohol (iPrOH) and the solution was acidified with 0.125 mL of concentrated HCl (for complete neutralization of Bu_4_NOH). Precipitation of a light-yellow solid started immediately and was completed within 2–3 h. The solid material was separated by centrifugation and then vacuum dried. A total of 149 mg of material was collected. This material was characterized by FT-IR (Fourier Transform Infra Red) spectroscopy, solid-state ^13^C NMR (Nuclear Magnetic Resonance) spectroscopy and thermal gravimetric analysis (Supplementary Figures 1–3). The synthesized material was concluded to have the brutto chemical formula C_60_(OH)_44_(6–8)H_2_O. A similar procedure was followed for C_70_ giving the brutto chemical formula C_70_(OH)_50_(14–16)H_2_O.

To make aqueous fullerol suspensions, fullerol powder was added to sterile M9 minimal media and dissolved by stirring. All fullerene and fullerol suspensions were stored in the dark before use. To prepare fullerene and fullerol suspensions for UV irradiation, 100 mg/L of C_60_, C_70_ or their respective fullerols were added to sterile M9 media and dispersed using the methods previously described, as appropriate. The resulting suspensions were purged under nitrogen and sealed inside quartz Erlenmeyer flasks inside an anaerobic chamber using rubber butyl stoppers and parafilm. The flasks were then removed from the chamber and placed 10 cm below a suspended UV lamp within a dark container. Suspensions were irradiated at 254 nm for 2 weeks to obtain the UV-irradiated suspensions used in subsequent culture experiments. UV-vis spectra of the suspensions were taken before and after irradiation and compared.

### 2.2 Fullerene aggregate sizing

Dynamic light scattering (DLS: Malvern ALV/LSE-5004) was used to determine the hydrodynamic diameters of the suspensions in distilled water and sterile M9 media. The results were also compared to diffusion measurements using differential dynamic microscopy (DDM). The influence of UV irradiation on the measured size distributions after 2 weeks, using conditions identical to those above, and after a further 2 weeks was determined.

### 2.3 Glassware

Fifteen-milliliter glass serum bottles and airtight butyl rubber stoppers were used for all microcosm culturing. To ensure all glassware and stoppers were sterile and free of organic contaminants, an adaptation of the protocol described by Eaton and Franson (2005) was used. Briefly, all glassware was washed with detergent (Decon90, 2% solution), rinsed three times with deionized water and soaked in 0.1 M HCl for a minimum of 12 h. The glassware was then rinsed again three times with deionized water, air dried, capped with aluminum foil and heated in a furnace at 550°C for 6 h. The butyl rubber stoppers were washed with detergent, boiled three times for 5 min in deionized water and air dried under a laminar flow hood.

### 2.4 Microorganisms

An environmental microbial community sample was collected from anaerobic pond sediment (Blackford pond, Edinburgh) and selectively cultured over three rounds of sub-culturing to grow on raw meteorite material by Waajen et al. (2022). The resulting anaerobic community was used in these experiments. An isolate from this community was obtained from a single colony, itself obtained by swabbing the surface of an M9 agar plate with the community described above supplemented with glucose. The selected isolate was sub-cultured three times to obtain a pure culture with uniform colonies.

### 2.5 Culture preparation

Microcosm volumes of 5 mL were used throughout the experiment and all conditions and controls were prepared in triplicate. All positive controls consisted of 5 mL of M9 minimal media supplemented with 0.4% (w/v) glucose. All conditions with fullerenes, fullerols or their derivatives were made with 5 mL of the suspension as described above either with or without the addition of 0.4% (w/v) glucose as specified. Negative controls were prepared as described above, but without microbial inoculation.

All microcosms were prepared aerobically under sterile conditions and then purged with N_2_ for 15 min to remove dissolved oxygen. After purging, the microcosms were moved into an anaerobic chamber (COY Laboratory Products Inc., vinyl anaerobic chamber 2% hydrogen 98% nitrogen) for the duration of the experiment.

All microcosms except negative controls were inoculated with 50 μL from a subculture of the anaerobic community or isolate in M9 minimal media with 0.4% (w/v) glucose.

### 2.6 Cell counting

Over a 28-day growth period, 100 μL aliquots of each microcosm were taken for direct cell counting measurements, beginning on day 0 immediately after inoculation and on subsequent days, as specified. Forty-five microliters of each aliquot was added to 5 mL of SYBR gold nucleic acid stain (Invitrogen) to a final working solution (diluted 1:1,000 in sterile deionized water) according to the manufacturer’s instructions for 5 min at room temperature. Ten microliters was pipetted into the chamber of a hemocytometer (Blaubrand Neubauer Bright-Line 0.100 mm × 0.0025 mm^2^). Cell counting was carried out under a 20x magnification microscope lens with a fluorescent light source (Leica DM 4000 B, Leica Kübler CODIX) and cell density was calculated.

### 2.7 Transmission electron microscopy

To obtain transmission electron microscopy (TEM) images of C_60_ cultures and controls, microcosms were prepared and grown following the previously described method. One milliliter of each culture was washed three times with 1x PBS buffer and resuspended in 1x PBS. Samples were fixed in 3% glutaraldehyde in 0.1 M sodium cacodylate buffer (pH 7.3) for 2 h and then washed three times in 0.1 M sodium cacodylate for 10 min. Each sample was post-fixed in 1% osmium tetroxide in 0.1 M sodium cacodylate for 45 min then washed three times in 0.1 M sodium cacodylate for 10 min. Samples were then dehydrated in 50, 70, 90, and 100% ethanol for 15 min each, then twice with propylene oxide for 10 min, before being embedded in TAAB 812 resin. One millimeter thick sections were cut on a Leica Ultracut ultramicrotome and stained with toluidine blue. Suitable areas for investigation were selected by viewing under a light microscope, and these areas were further cut into 60 nm ultrathin sections and stained with uranyl acetate and lead citrate. Samples were viewed with a JEOL JEM-1400 Plus TEM and images were collected on a GATAN OneView camera. The material observed in the TEM images was confirmed with Raman spectroscopy (Renishaw) to be intact C_60_ cages using the same ultrathin section samples (785 nm, 1 mW, 10 s exposure time) (Supplementary Figure 5).

### 2.8 Microbial growth analysis

For each microcosm, the final cell density counted on day 28 was converted to a percentage increase relative to the initial day 0 cell count. This was done to allow direct comparison of cell densities between conditions while accounting for the inevitable discrepancy in initial cell number immediately after inoculation. All subsequent analysis was performed on the cell density percentage increase values, rather than the raw cell density. The average cell density percentage increase was used for graphical representation.

To compare each condition to the control or another condition, an *F*-test was performed to determine if the variances between the two conditions were equal or unequal. *t*-tests assuming unequal or equal variances, as appropriate, were performed assuming a significance level of α = 0.05. In all graphical representations, a “*” is used to denote a significant result while “ns” represents a non-significant result, usually when compared to the positive control, unless otherwise specified. All error bars represent the standard error on the mean for each condition.

### 2.9 DNA sequencing and microbial identification

Cultures were prepared for DNA extraction by inoculating 10 mL of anoxic M9 media and 0.4% (w/v) glucose with either the anaerobic community or the isolate from single plated colonies. These were grown anaerobically for 28 days as previously described. DNA was extracted from each culture using an extraction kit (DNeasy PowerSoil Pro, QIAGEN) per the manufacturer’s instructions and quantified using a Qubit 3 fluorometer (Invitrogen). DNA samples were sent to Novogene, United Kingdom, for Illumina 16S rDNA sequencing and bioinformatics analysis. The following methods were carried out by Novogene. The 16S rDNA variable region (V3-4) region was amplified via PCR using the primers CCTAYGGGRBGCASCAG and GGACTACNNGGGTATCTAAT. The resulting amplicons were sequenced on an Illumina NovaSeq 6000 paired-end platform to generate 250 bp reads. The raw amplicon data was cleaned using DADA2 (RRID:SCR_023519) software to remove noise and obtain non-duplicated amplicon sequence variants (ASVs). QIIME2 (RRID:SCR_021258) software was used to annotate each ASV based on the Silva 138.1 open source database. From this data, species abundance tables were generated at the level of kingdom down to genus for the anaerobic microbial community.

## 3 Results

### 3.1 Growth of anaerobic community in the presence of fullerenes and derivatives

**FIGURE 1:**
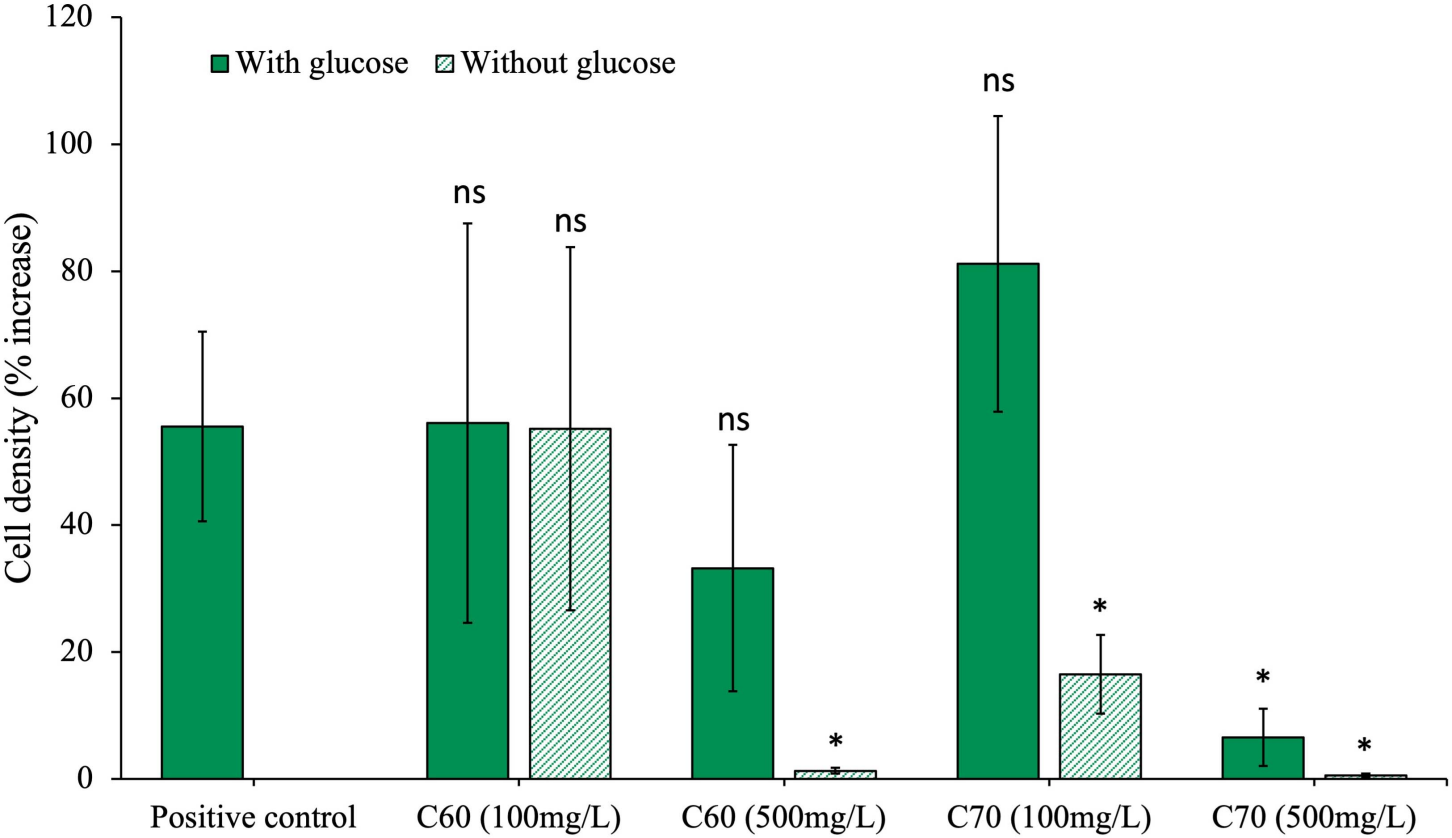
Effects of C_60_ and C_70_ on anaerobic community growth. The community was cultured with either a low (100 mg/L) or high (500 mg/L) concentration of C_60_ or C_70_, with and without additional glucose. Final cell density counted on day 28 of growth and expressed as a percentage increase from day 0 post-inoculation. Error bars represent the standard error on the mean for each condition. All statistics are carried out with either paired or unpaired *t-*tests (dependent on the significance of an *F*-test) comparing each condition to the positive control where *p* > 0.05 is considered significant.

The anaerobic community was grown for 28 days with two different concentrations of C_60_ or C_70_; a concentration of 100 mg/L or a higher one of 500 mg/L (Figure 1). The community was grown with either C_60_ or C_70_ as the sole carbon source or with additional glucose to test whether either fullerene would have an inhibitory effect on the growth of the bacteria on other carbon sources. The results were compared to the positive control using an unpaired *t*-test with a significance level of 0.05, and the *p*-values obtained are listed in Table 1. The results indicate that a concentration of 100 mg/L C_60_ did not significantly change the growth of the community compared to the positive control, regardless of whether glucose was present or not. There was no significant difference between the C_60_ (100 mg/L) cultures with or without glucose (*p* = 0.094) which suggests that in this experiment, C_60_ (100 mg/L) in the presence of glucose did not increase the amount of growth, but in the absence of glucose, the C_60_ alone could be used as a carbon source.

**TABLE 1:** Significance of the effects of anaerobic community growth with C_60_ and C_70_ at 100 mg/L or 500 mg/L, with and without glucose, compared to the positive control (glucose).

Condition	*P*-value
C_60_ (100 mg/L) with glucose	0.99
C_60_ (100 mg/L) without glucose	0.99
C_60_ (500 mg/L) with glucose	0.41
C_60_ (500 mg/L) without glucose	0.03
C_70_ (100 mg/L) with glucose	0.42
C_70_ (100 mg/L) without glucose	0.04
C_70_ (500 mg/L) with glucose	0.04
C_70_ (500 mg/L) without glucose	0.03

At a concentration of 500 mg/L, C_60_ did not significantly affect community growth compared with the positive control, indicating that it was not inhibitory at this concentration provided there was an additional carbon source. However, when there was no glucose, there was no notable growth (cell density was indistinguishable from the negative control), which suggests that at the higher concentration, C_60_ could no longer be used as a carbon source.

Community growth with C_70_ at 100 mg/L was not significantly different from the positive control if glucose was available, however, if no glucose was present, the cell density was much lower. At 500 mg/L, community growth with C_70_ was significantly reduced both with and without glucose, indicating that at the higher concentration, C_70_ could not be used as a carbon source and the low level of growth recorded in the presence of glucose compared to the positive control suggests that it was inhibitory to growth.

We then sought to test the reproducibility of the result which indicates the community could use C_60_ as a carbon source. We did this by conducting four different growth experiments in which the community was cultured with C_60_ (100 mg/L) as a sole carbon source. The results are summarized in Supplementary Figure 4. For each experiment, the cell density (% increase) of the C_60_ condition on day 28 was compared to the corresponding positive control. In experiments 1–3, growth with C_60_ as a sole carbon source was significantly lower than the positive controls (*p* = 0.021, 0.02 and 0.05 for experiments 1–3, respectively), which suggests that although growth was reduced compared with the positive control, there was a small amount of C_60_ use occurring. Conversely, in experiment 4, there was no significant difference in cell density between the C_60_ condition and the positive control (p = 0.99), indicating growth was very similar with C_60_ as a carbon source compared with the glucose in the positive control.

**FIGURE 2:**
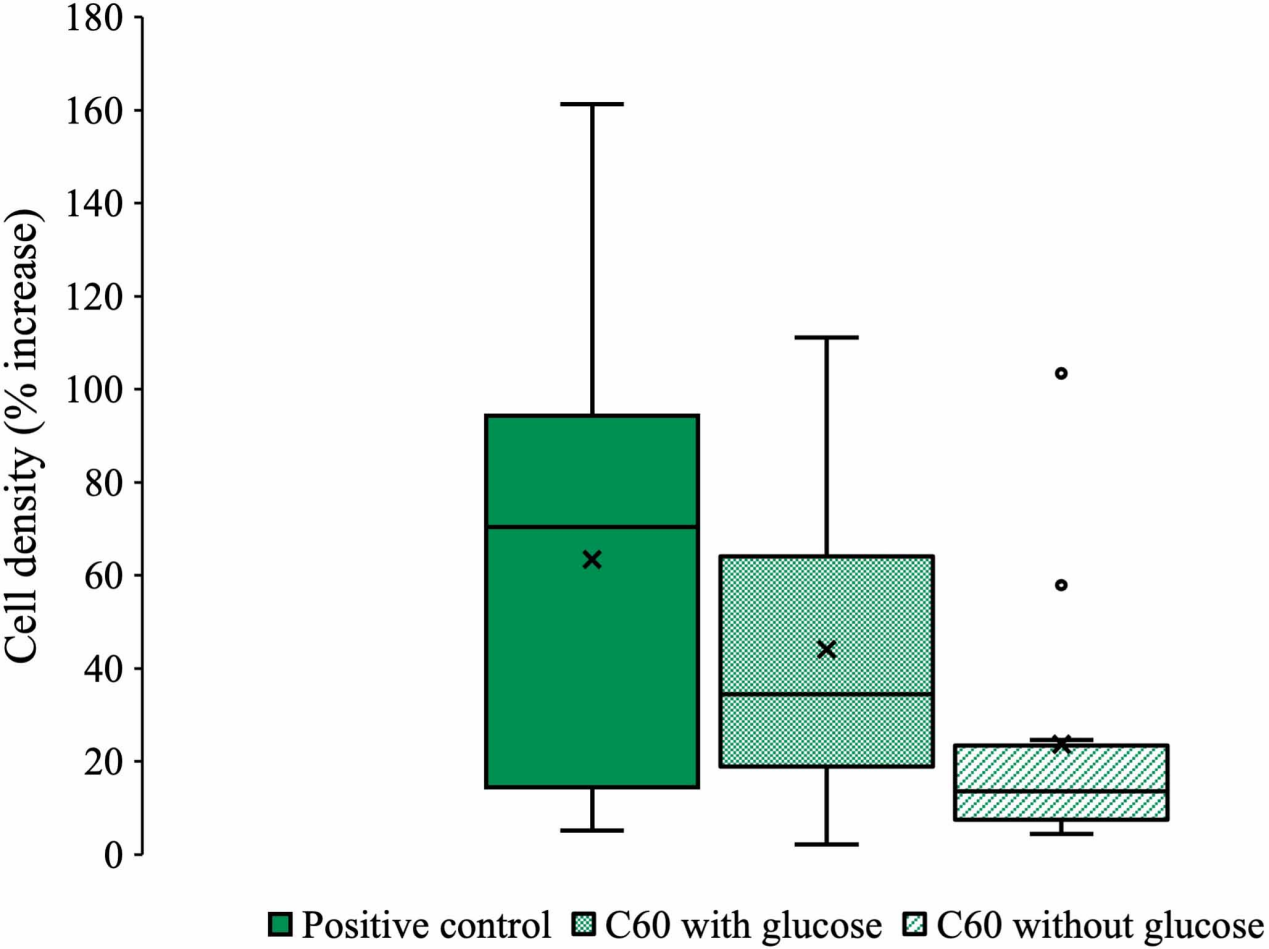
Collected microbial community growth data from experiments with C_60_. Box and whisker plot compiling all the pooled data from each experiment where the anaerobic community was cultured with C_60_ (100 mg/L) with or without additional glucose. Plot whiskers describe the range of data points for each condition, the outer bounds of the solid boxes represent the lower and upper quartiles, the middle line is the median and X markers represent the mean. Outliers are displayed as open circles.

Figure 2 summarizes all the data from experiments in which the community was cultured with C_60_ (100 mg/L) as a sole carbon source or with additional glucose. The box and whisker style plot gives an indication of how the final cell densities of each condition overlap as well as allowing for comparison of the mean and average spread of the data. The C_60_ with glucose condition consistently overlapped with the positive control, showing that when glucose was available, the presence of C_60_ had little influence on growth. Growth with C_60_ as a sole carbon source was lower compared to the positive control, except for two outliers in which the final cell densities were as high. However, some growth was observed, which suggests it can be used as a carbon source.

**FIGURE 3:**
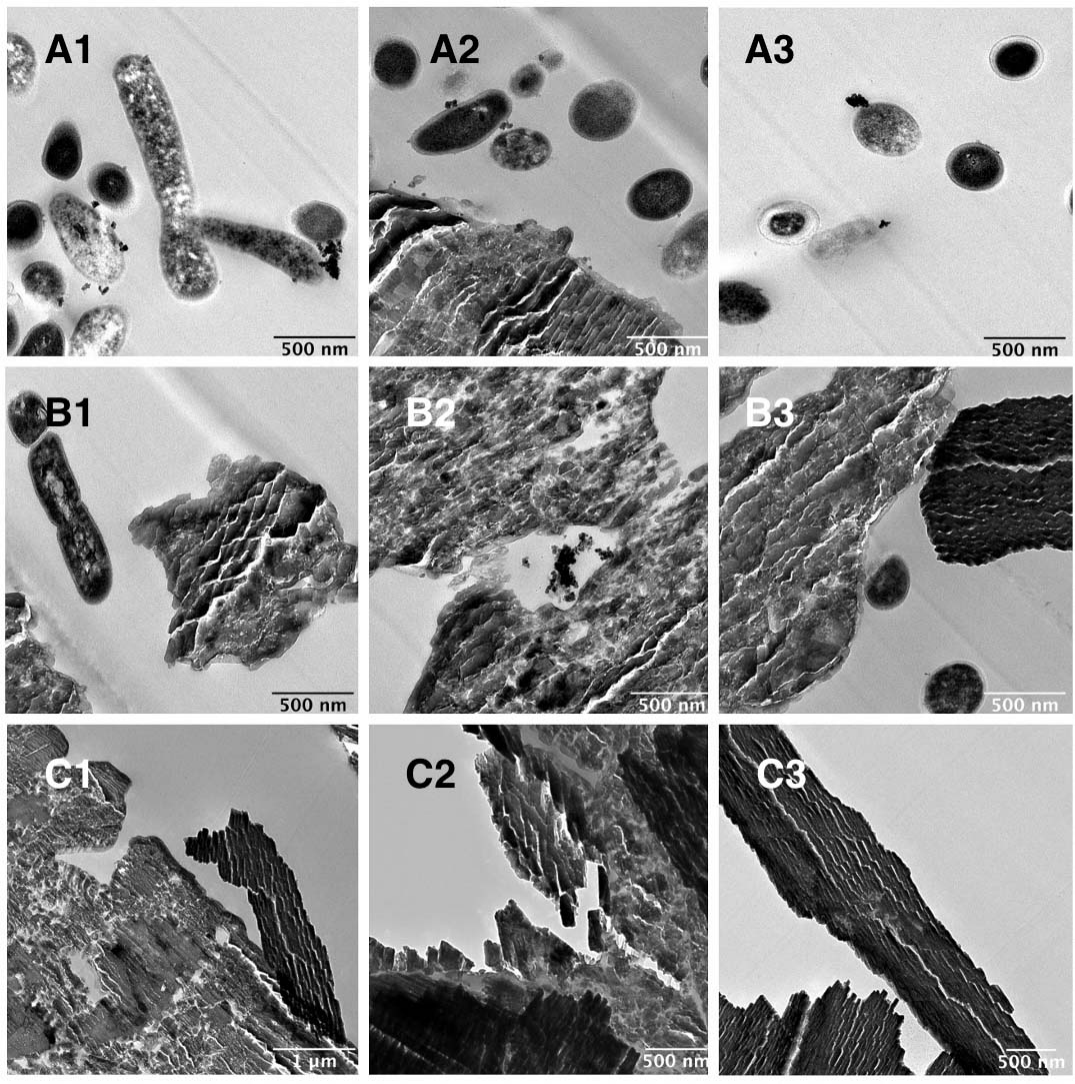
TEM images of anaerobic community cultures grown with 100 mg/L C_60_ as a sole carbon source or with glucose. **(A)** Community grown with 100 mg/L C_60_ as a sole carbon source, **(B)** community grown with 100 mg/L C_60_ with additional glucose, **(C)** 100 mg/L C_60_ negative controls (not inoculated).

The anaerobic community was cultured with 100 mg/L C_60_ with or without additional glucose and TEM images were obtained of these cultures on day 28 of growth (Figure 3). Figure 3A shows images of the community grown with C_60_ as a sole carbon source, where the aggregated material was confirmed by Raman spectroscopy to be C_60_ with possibly some evidence for cage oxidation at the surface (Supplementary Figure 5), in agreement with previous observations of surface oxidation of C_60_ aggregates (Hwang and Li, 2010; Zygouri et al., 2020). In these images, small black deposits were observed on the surface of some of the bacterial cells. Figure 3B shows the community grown with C_60_ and additional carbon. In these images, aggregated C_60_ was observed but the small black cell surface deposits were not, suggesting these only occur in cultures in which C_60_ is the sole carbon source. Images of the negative controls (100 mg/L C_60_ with no bacteria) are shown in Figure 3C, where aggregated C_60_ was observed, and no bacterial cells were present.

DNA was extracted from the community and the 16S rDNA gene was amplified and sequenced to obtain the relative abundances of each taxon present in the community after incubation with C_60_ as the sole carbon source. [Supplementary Figure 6 shows the relative abundances of each taxonomic family detected. The majority of the species present belong to the Enterobacteriacaea family (82%)].

**FIGURE 4:**
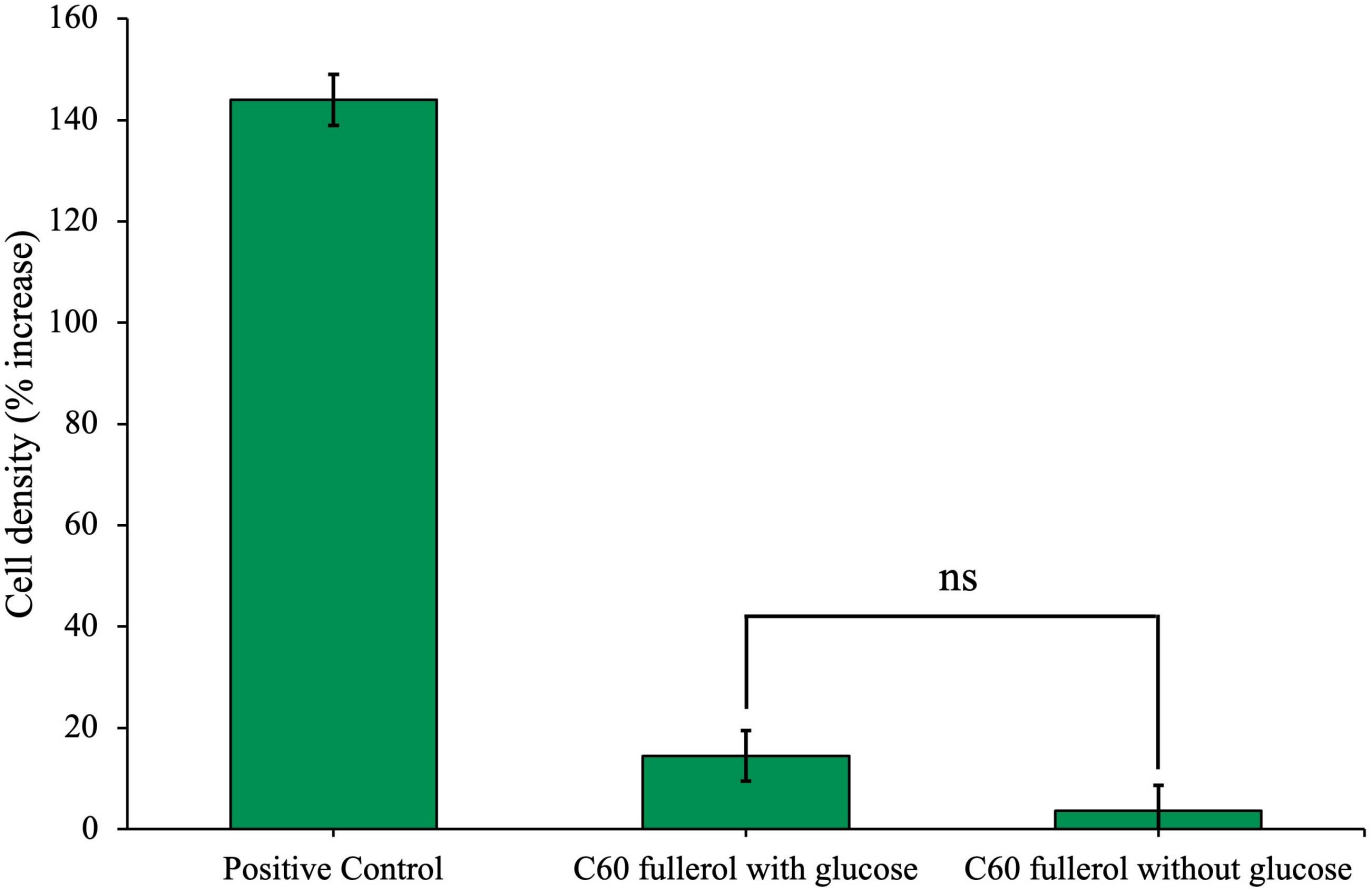
Effects of C_60_ fullerol on anaerobic microbial growth. Anaerobic community cultured for 28 days with 100 mg/L C_60_ fullerol, with or without glucose. Final cell density counted on day 28 of growth and expressed as a percentage increase from day 0 post-inoculation.

In addition to C_60_, we sought to investigate the effects of soluble fullerene derivatives on the anaerobic community. We achieved this by first culturing the community with 100 mg/L of the water-soluble C_60_ derivative, C_60_ fullerol, with and without additional glucose (Figure 4).

Community cell density with C_60_ fullerol was significantly reduced compared with the positive control on day 28, both with and without glucose (*p* = 0.046 and 0.042), and there was no significant difference between the C_60_ fullerol conditions with or without glucose (*p* = 0.092). The growth reduction in the presence of glucose compared to the positive control suggests that exposure to C_60_ fullerol at 100 mg/L under ambient conditions was inhibitory to the community. The small amount of growth without glucose could be attributable to use of the fullerol as a sole carbon source (although this could be potentially caused by carbon carry over as discussed later).

**FIGURE 5:**
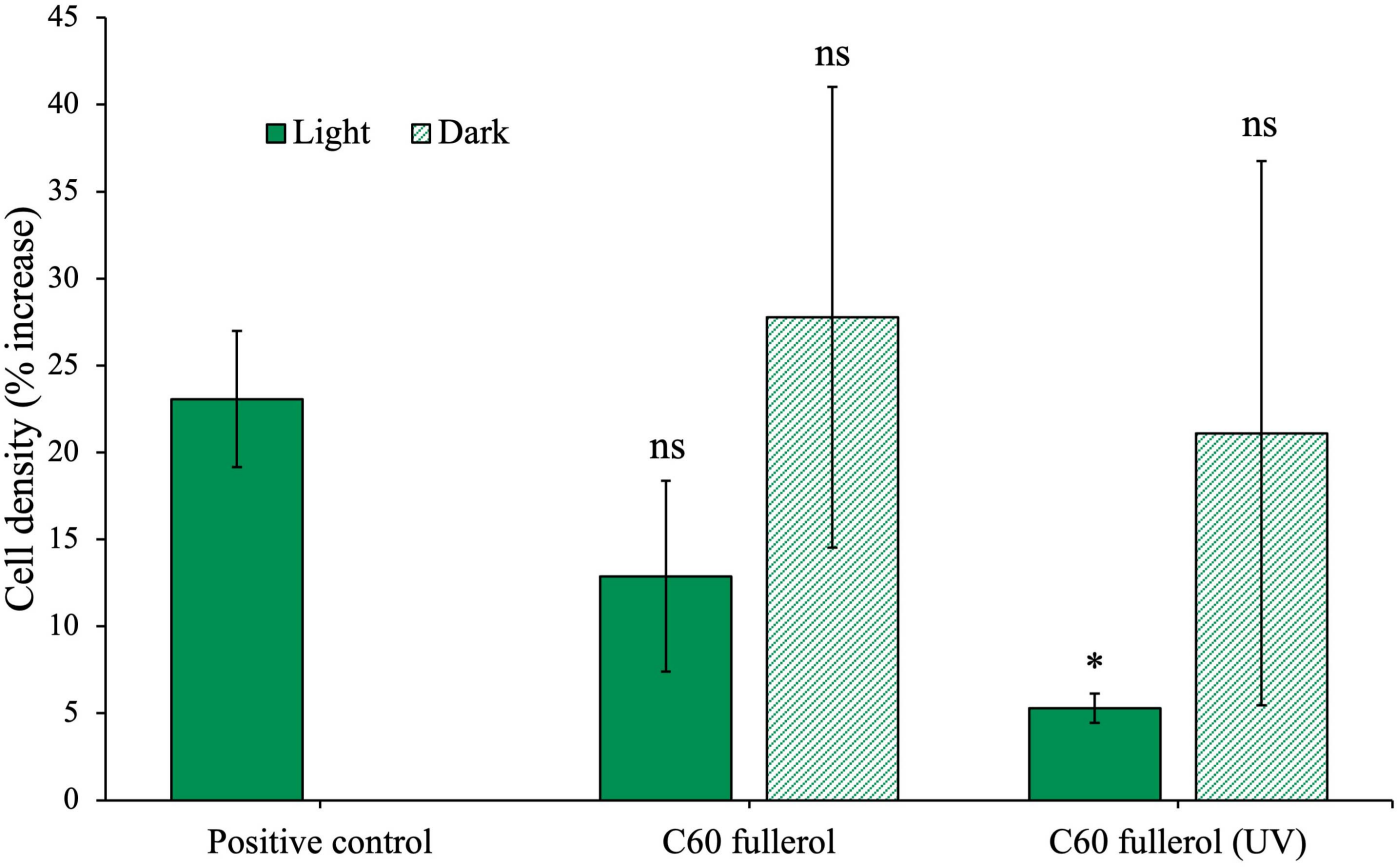
Effects of fullerols on the growth of the anaerobic community with or without prior UV exposure, in ambient light or dark growth conditions without glucose. Anaerobic community cultured for 28 days with 100 mg/L C_60_ fullerol. Final cell density counted on day 28 of growth and expressed as a percentage increase from day 0 post-inoculation. In the case of the positive control, since there was no effect of light, the growth data are compared to the light control, shown here.

C_60_ fullerol at a concentration of 100 mg/L was exposed to 254 nm UV radiation for 2 weeks before being used as a substrate for microbial inoculation. The community cultures were incubated for 28 days with C_60_ fullerol, both irradiated and non-irradiated, without glucose, under ambient lab light conditions as well as in the dark. The resulting cell densities are presented in Figure 5.

On day 28 of this experiment, we found that the non-irradiated C_60_ fullerol experiment (light or dark) did not have different cell densities compared to the positive control (light and dark conditions with *p*-values of 0.17 and 0.75, respectively). Note the difference in this result compared to the growth in fullerols in the experiment reported in Figure 4 in which cell concentrations were significantly lower compared to the positive control without glucose. We found no significant difference between the cultures incubated in the dark with UV-irradiated C_60_ fullerol and the positive control (*p* = 0.59). However, when the UV-irradiated C_60_ fullerol cultures were exposed to light, their growth was significantly reduced compared to the positive control (*p* = 0.005). Based on this, we infer that C_60_ fullerol is most toxic to the community when it has been irradiated with UV and is then subject to continued ambient light exposure after inoculation.

### 3.2 Growth of anaerobic isolate in the presence of fullerenes and derivatives

To determine if similar results would be obtained with an individual species, we repeated some of the community experiments with an anaerobic isolate obtained from the anaerobic community. The DNA sequencing results indicate that this isolate is from the family *Enterobacteriaceae*, however, due to difficulties with DNA extraction and sequencing we are unable to conclusively determine genus-level taxonomic identification.

**FIGURE 6:**
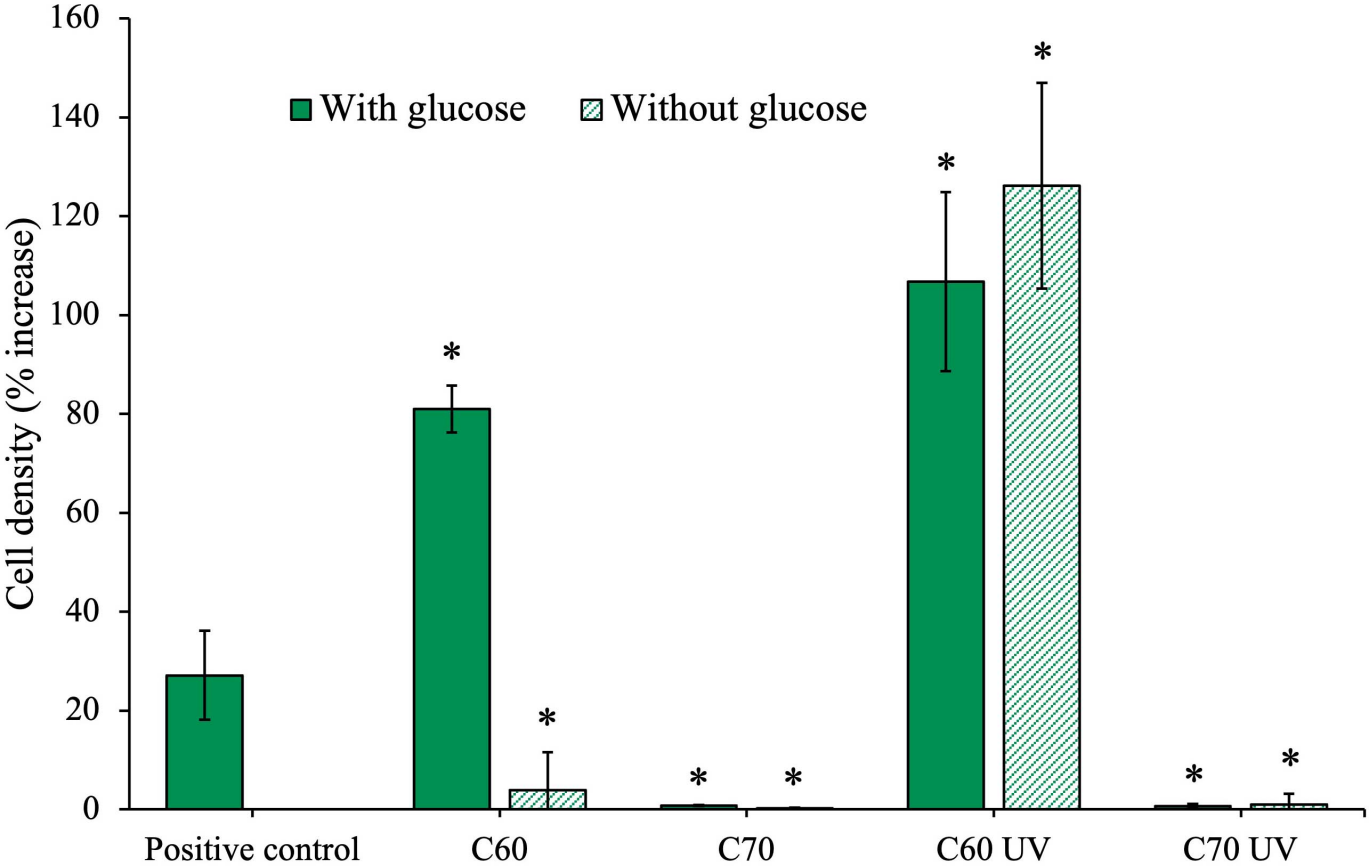
Effects of irradiated and non-irradiated fullerenes on growth of an anaerobic isolate. The isolate was cultured with C_60_ and C_70_ either in the native form or after UV irradiation, with and without additional glucose. Cell density (%) increase was measured on day 28 of growth. C_60_ and C_70_ UV were irradiated for 2 weeks before inoculation.

The isolate was grown for 28 days with 100 mg/L of native C_60_ or C_70_ or UV-irradiated C_60_ or C_70_, with or without additional glucose, the results of which are shown in Figure 6. Unpaired *t*-tests were performed to compare each condition to the positive control on day 28, the results of which are summarized in Table 2.

**TABLE 2:** Significance of anaerobic isolate growth with C_60_, C_70_ and their UV-degradation products, with and without glucose, compared to the positive control (glucose).

Condition	*P*-value
C_60_ with glucose	0.0003
C_60_ without glucose	0.04
C_70_ with glucose	0.03
C_70_ without glucose	0.01
C_60_ UV with glucose	0.01
C_60_ UV without glucose	0.01
C_70_ UV with glucose	0.01
C_70_ UV without glucose	0.01

The final cell density was increased compared to the positive control when the isolate was grown with C_60_ and additional glucose. This could suggest that C_60_ was being used in addition to glucose, resulting in the observed increase in growth. However, when glucose was not available, the isolate cell density was much lower and cannot be concludively attributed to use of the compound as a sole carbon source. Conversely, in cultures grown with UV-irradiated C_60_, we observed significant growth, greater than the positive control, even when no additional glucose is available. This suggests that the C_60_ UV degradation products produced after 2 weeks of irradiation were available as a carbon source.

The isolate did not grow in the presence of C_70_ at 100 mg/mL either with or without glucose, indicating that not only is the C_70_ not used, but it was also highly inhibitory. Similarly, isolate growth was severely inhibited in both C_70_ UV conditions, indicating the UV degradation products of C_70_ were also highly inhibitory to growth in the isolate.

**FIGURE 7:**
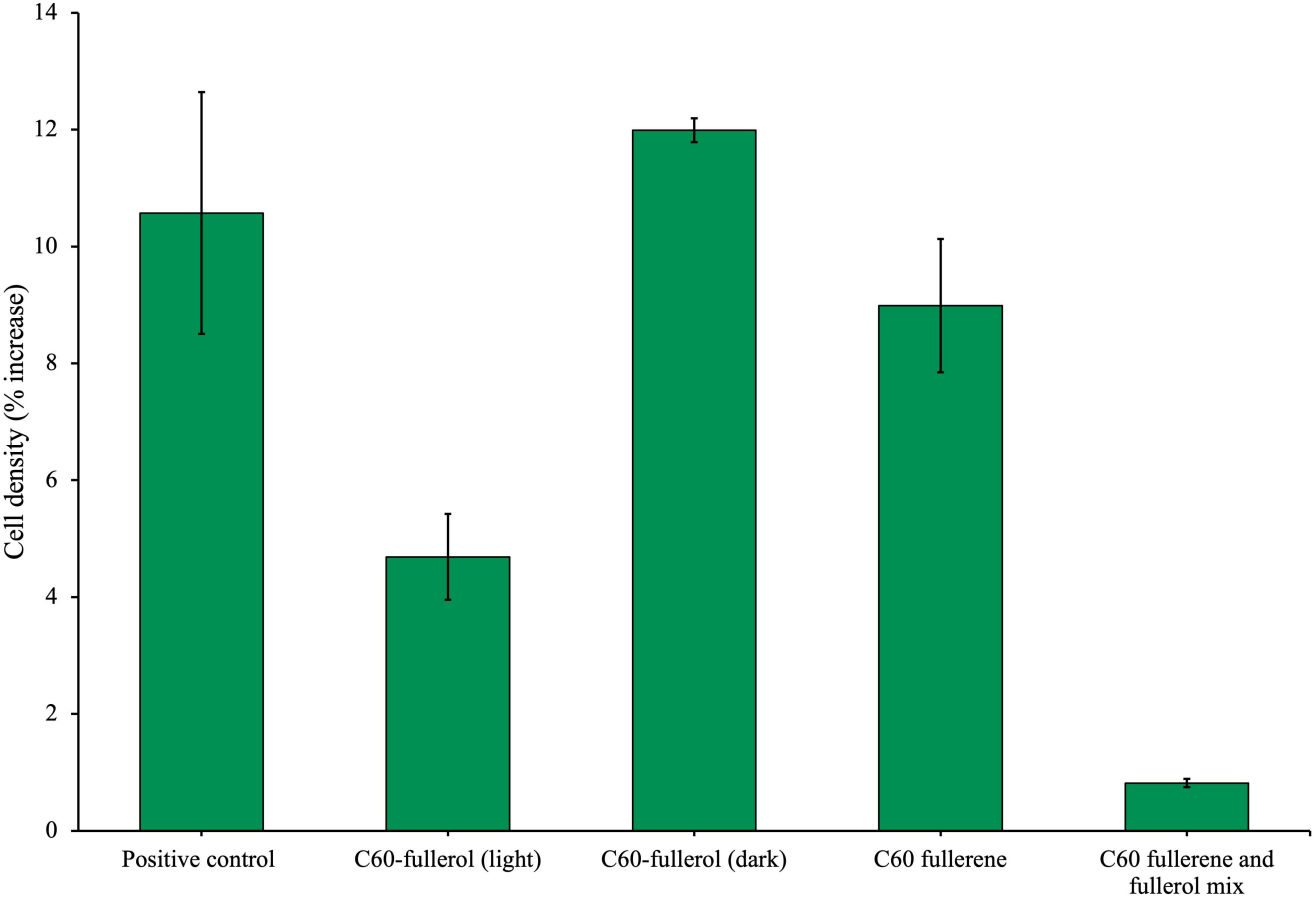
Effects of fullerols and fullerene/fullerol mixture on the growth of an anaerobic isolate. The isolate was cultured with either C_60_ fullerol (100 mg/L) in the light or dark, C_60_ fullerene (100 mg/L) or a 100 mg/L equal mix of C_60_ fullerene and fullerol. All conditions were supplemented with glucose. Cell density (%) increase was measured on day 28 of growth. C_60_ and C_70_ UV were irradiated for 2 weeks before inoculation.

As we did with the anaerobic community, we next investigated the growth of the isolate with fullerol derivatives. The isolate was cultured for 28 days with 100 mg/L C_60_ fullerol under two different conditions: in ambient lab light or the dark, and this was compared to growth with the C_60_ fullerene (100 mg/L) and a mixture of C_60_ fullerene and fullerol in a total combined concentration 100 mg/L (Figure 7). All cultures were given additional glucose. The results show that when grown with C_60_ fullerol in the light, the cell densities on day 28 were significantly lower compared to the positive control (*p* = 0.043). However, when grown in the dark, there was no significant difference from the positive control (*p* = 0.47). These findings suggest that C_60_ fullerol was more inhibitory to the isolate when exposed to light, qualitatively similar to the results obtained with the community.

We found that when C_60_ fullerol and fullerene were mixed, the mixture was inhibitory to the isolate in the presence of glucose (Figure 7), as it significantly reduced growth compared with the positive control (*p* = 0.0095). For comparison, the cultures grown with C_60_ in the presence of glucose were not significantly different to the positive control (*p* = 0.68), which suggests it is the presence of the fullerol in this mixture which caused it to be inhibitory.

**FIGURE 8:**
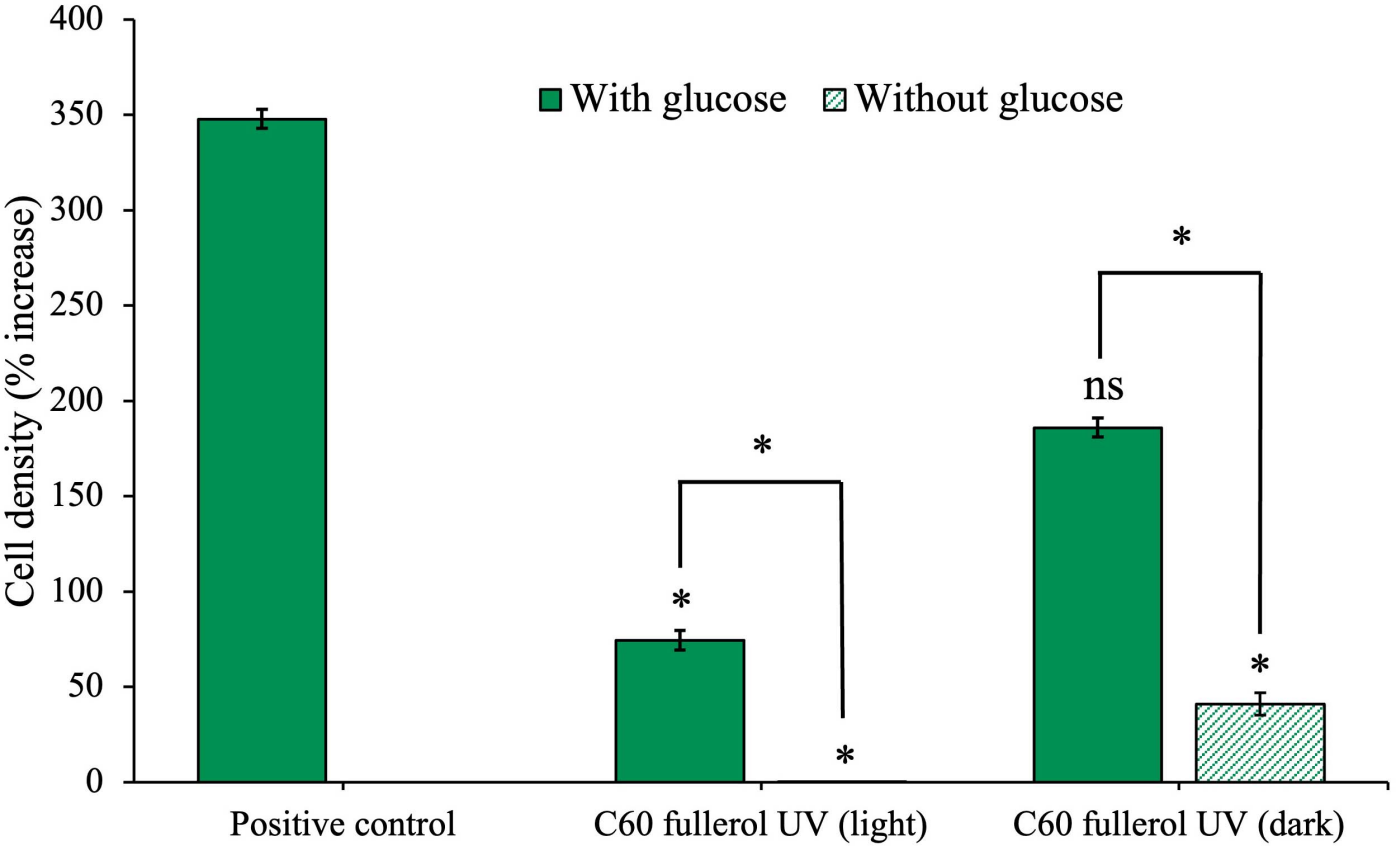
Effects of UV-irradiated C_60_-fullerol on the growth of an anaerobic isolate. Cell density after 28 days of growth with UV-irradiated C_60_ fullerol in light or dark growth conditions, with and without supplementary glucose is shown.

Our next objective was to investigate how UV radiation exposure impacts the effect of C_60_ fullerol on the anaerobic isolate. To achieve this, we exposed C_60_ fullerol suspensions to 254 nm UV radiation for 2 weeks. We then cultured the isolate with the irradiated fullerol under two conditions: in the dark and under ambient light, both with and without glucose (Figure 8).

The culture with glucose and UV-irradiated C_60_ fullerol in ambient light (Figure 8) had significantly reduced growth compared to the positive control (*p* = 0.05) indicating that the C_60_ fullerol was inhibitory after UV irradiation and with sustained ambient light exposure. Without glucose, growth was negligible (*p* = 0.04 compared to the positive control), which may be attributable to the lack of a carbon source for growth (both a lack of glucose and the inability to use UV-irradiated C_60_ fullerol as a carbon source). There was a significant difference between the light conditions with and without glucose (*p* = 0.0073), indicating that although the UV-irradiated C_60_ fullerol was inhibitory in the light, there was still growth occurring when glucose was available.

In contrast, under dark conditions (Figure 8), we observed that if glucose was available, isolate growth with UV-irradiated C_60_ fullerol was not significantly different to the positive control (*p* = 0.1) suggesting that the UV-irradiated C_60_ fullerol was not inhibitory in the dark provided it was kept in the dark. However, there was significantly reduced growth when no glucose was present (*p* = 0.045) which could suggest some use of the UV-irradiated products of C_60_ fullerol as a sole carbon source, noting the caveats discussed later. These results suggest that the highest toxicity of UV-irradiated C_60_ fullerol was linked to prolonged light exposure.

**FIGURE 9:**
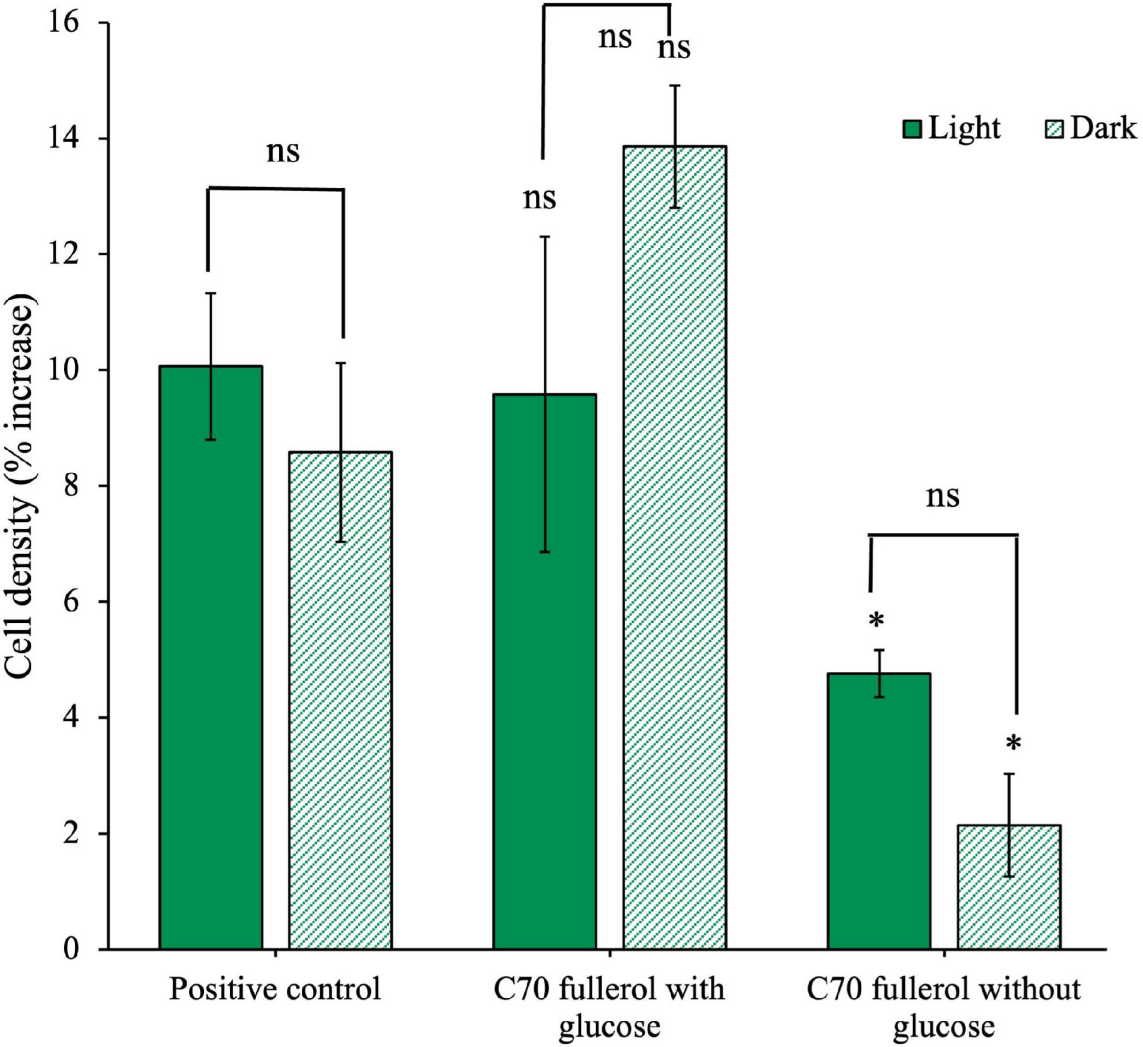
Effects of C_70_ fullerol on the growth of an anaerobic isolate. Cell density after 28 days of growth with C_70_-fullerol, with and without glucose under light or dark growth conditions.

We next sought to investigate how light conditions could influence the growth of the anaerobic isolate in the presence of the larger C_70_ fullerol. The isolate was grown with 100 mg/L C_70_ fullerol in either ambient light or dark conditions, as a sole carbon source or with additional glucose (Figure 9). In this experiment, the positive control was also kept in either light or dark conditions, however because there was no significant difference between growth in the light or dark (*p* = 0.5), all statistical comparisons were made using the cell density of the light condition.

We found no significant difference between either of the C_70_ fullerol conditions with glucose and the positive control (*p* = 0.88 and 0.82 for the light and dark conditions, respectively). Additionally, we found there was no significant difference between the C_70_ fullerol with glucose in light and dark conditions (*p* = 0.22), all of which indicate that as long as glucose is available, the C_70_ fullerol was not inhibitory and that exposure to light made no significant difference. When glucose was not available, however, growth in both the light and dark C_70_ fullerol conditions was significantly reduced compared to the positive control (*p* = 0.029 and 0.0068 for light and dark, respectively). Without glucose, growth was significantly less than the positive control. In this experiment, overall growth in all conditions was small (not exceeding 15% of cell starting concentrations) and thus should be treated with caution.

### 3.3 Fullerene particle sizing

**FIGURE 10:**
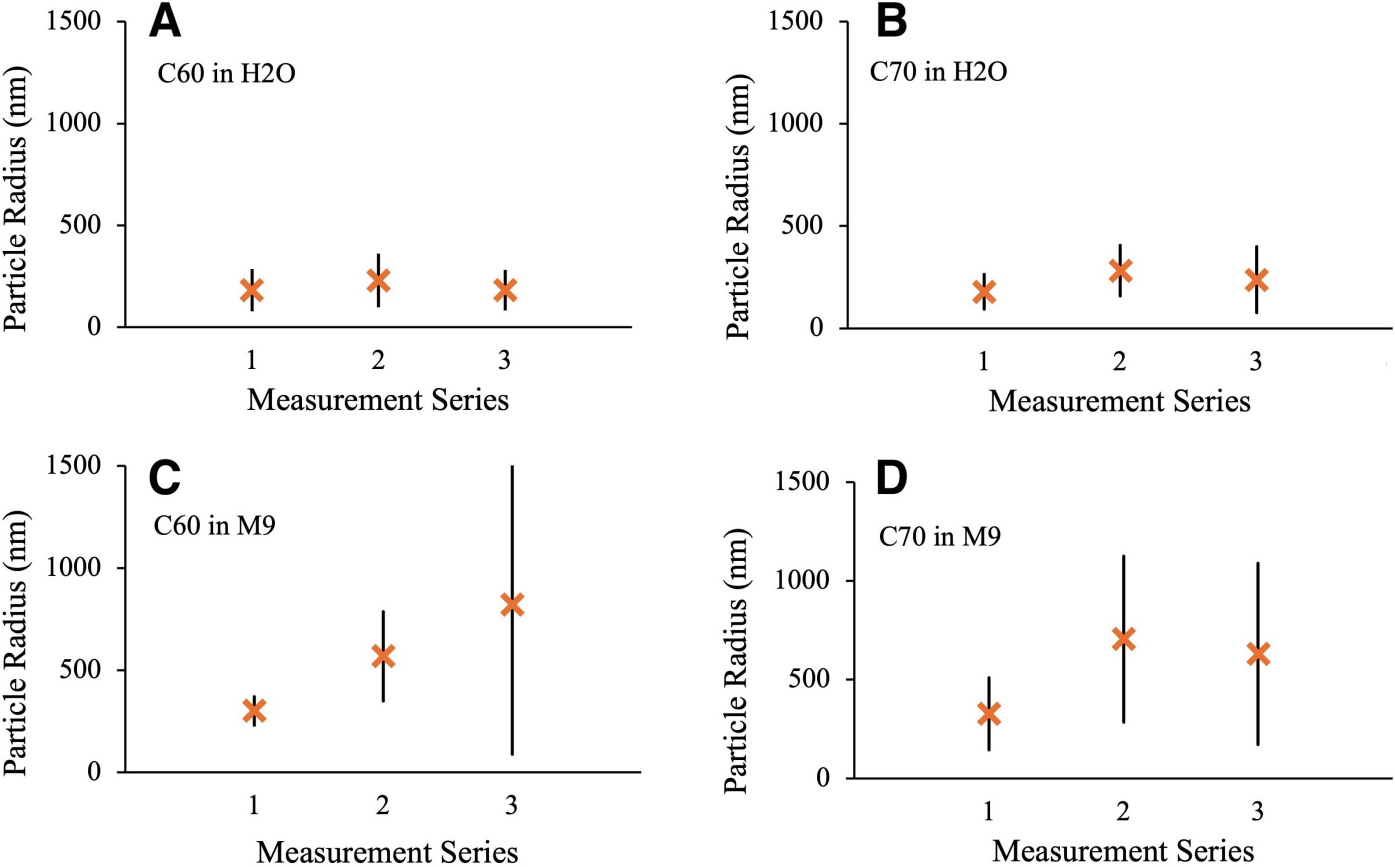
DLS measurements of aggregate radius. Series 1: fresh suspension, Series 2: After 2 weeks UV irradiation, Series 3: after 4 weeks UV irradiation. **(A)** 100 mg/L C_60_ in distilled H_2_O **(B)** 100 mg/L C_70_ in distilled H_2_O **(C)** 100 mg/L C_60_ in M9 **(D)** 100 mg/L C_70_ in M9.

UV-vis spectra and DLS measurements were taken of C_60_ and C_70_ suspensions in either distilled water or M9 media just after preparation, after 2 weeks of UV irradiation and after 4 weeks of UV irradiation. The results of the DLS measurements can be seen in Figure 10 where the limits indicate the spread of size distributions for each condition. DLS measurements indicate the presence of large aggregates, particularly in the M9 media, which confirms the observations made in the TEM images in Figure 3. In our suspensions, there was a greater propensity for large aggregate formation in M9 media compared to distilled water for both C_60_ and C_70_, which is in agreement with previous studies (Kyzyma et al., 2019). The initial aggregate sizes of both C_60_ and C_70_ in freshly prepared H_2_O suspensions (measurement series 1) were very similar (approximately 180 ± 100 nm hydrodynamic radius). In M9, the aggregate sizes are also similar to each other with 300 ± 70 nm and 330 ± 180 nm for C_60_ and C_70_, respectively.

**FIGURE 11:**
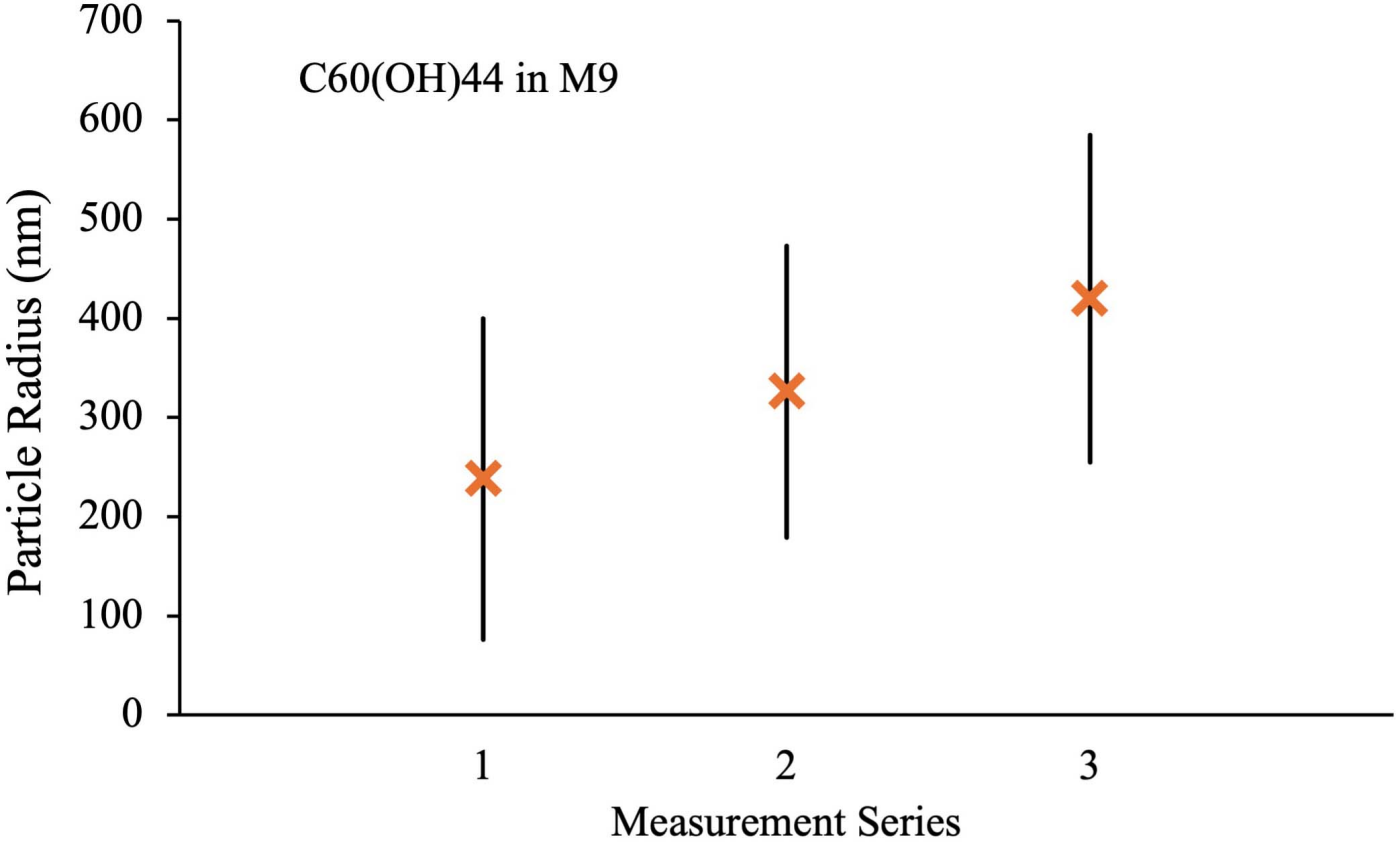
DLS measurements for C_60_(OH)_44_ in M9 media. Series 1: fresh suspension, Series 2: After 2 weeks UV irradiation, Series 3: after 4 weeks UV irradiation.

Measurement series 2 was obtained after 2 weeks of UV exposure under conditions identical to those used for the microbial growth studies. In all cases, there was an increase in the size of the aggregates after UV irradiation, most notably for C_60_ and C_70_ in M9 where the mean radii increase to 570 ± 220 and 700 ± 420 nm, respectively. Doubling the UV exposure time to 4 weeks (measurement series 3) made very little difference to the particle radii and distributions for either H_2_O suspension or for C_70_ in M9, if anything slightly reducing the values. For C_60_ in M9, however, the mean radius and size range continued to increase with longer UV exposure to 820 ± 720 nm. Corresponding results for the C_60_ fullerol in M9 are shown in Figure 11. Here, the aggregate sizes in M9 before UV irradiation were somewhat smaller than for the non-functionalized fullerenes, 240 ± 160 nm with a smaller increase in aggregate size on irradiation to 330 ± 150 nm and 420 ± 160 nm after 2 and 4 weeks, respectively.

## 4 Discussion

### 4.1 Growth of C_60_ fullerenes with anaerobic microorganisms

With this study, we aimed to evaluate the interactions of fullerenes and their derivatives with modern anaerobic microorganisms for a better understanding of how these materials may have been used as a carbon source by early anaerobic life on Earth and other young planets receiving an extraterrestrial influx of organic materials.

Our initial experiments were conducted using an anaerobic community that had already been selected to grow on powdered meteorite containing a representative sample of the types of organics likely to have been present on the early Earth (Waajen et al., 2022). The community, originally derived from anaerobic pond sediment, was chosen for its environmental relevance as well as its predisposition to dealing with complex organic mixtures. In the original study by Waajen et al., a reduction in diversity was observed after the community was grown with powdered meteorite material, indicating that habitat filtering was taking place to produce a more specialized community. Growth with meteorite material created a niche that selected for certain species present in the original community, favoring species from the Deltaproteobacteria, Geobacteraceae and Desulfuromonadaceae families which are known to use sulfur and iron as electron acceptors (Waajen et al., 2022). In our experiments, we observed a further decrease in diversity when this community was grown with fullerene C_60_ as the carbon source. This is in line with the habitat filtering observed by Waajen et al., as by limiting the carbon source to C_60_, we created an even narrower niche for microbes to grow, increasing habitat selection pressure which results in fewer species present. Interestingly, we found that the majority of the species present in the community after growth with C_60_ belong to the Enterobacteriaceae family. Although this is a large family consisting of over 30 genera such as *Escherichia*, *Klebsiella* and *Shigella*, most are only facultative anaerobes. Other families present in the C_60_-adapted community were from the taxonomic families *Pleomorphomonadaceae, Cellulomonadaceae, Xanthomonadaceae, Microbacteriacea* and *Rhizobiaceae*, all of which are made up primarily of environmentally associated species found in soils.

We observed the anaerobic community to grow in the presence of only C_60_, suggesting that it may use this as a sole carbon source. However, in our four non-simultaneous growth experiments where the community was given 100 mg/L C_60_ as the sole carbon source, we found that the degree to which the C_60_ cultures grew compared to the positive controls varied between these seemingly identical experiments. There was consistently at least some degree of growth observed with C_60_ as the sole carbon source, and in some cases, growth was comparable to that of the community with glucose. We suggest that this discrepancy between experiment repeats might be caused by a shift in community composition over time arising from its complex microbial structure, even though starting conditions were similar. Similar variations in growth compared to positive controls were observed between ostensibly identical conditions in experiments involving fullerols.

With respect to growth on C_60_, we cannot completely rule out trace carbon in reagents that allowed for the growth of cells in C_60_. However, both Raman and FTIR spectroscopic analysis of dried M9 medium preparations did not reveal any contaminant carbon (data not shown) and glassware was acid washed and baked (see Materials and Methods). There is the potential for some carry over of organic carbon from the inoculum and we cannot rule out intracellular carbon reserves in cells which could allow for growth. However, given that growth with C_60_ (100 mg/L) in some of the experiments was not significantly different from positive control growth with glucose, we attribute these results to the potential use of C_60_ as a carbon source. Nevertheless, in all our experiments where low levels of growth were observed in fullerenes or fullerols and their derivatives in the absence of glucose, caution should be excercised in interpreting the use of these compounds as sole carbon sources. Isotopically labeled tracer experiments, microcalorimetry or “omics” approaches could be used to more directly investigate metabolic use of the compounds.

At the higher C_60_ concentration of 500 mg/L, although culture growth was the same as the positive control if supplementary glucose was available, it was significantly reduced when this additional carbon source was removed, and much lower than the growth obtained under similar conditions with 100 mg/L C_60_ (without glucose) suggesting that at these higher concentrations an inhibitory effect may be occurring which can be overcome if glucose is provided as a substrate. This result might be consistent with previous studies in which C_60_ was shown to reduce the growth of *Pseudomonas fluorescens* proportionally to the concentration of C_60_, meaning higher concentrations were more inhibitory (Riding et al., 2012).

Previous studies that have assessed the effect of C_60_ on anaerobic microorganisms are very limited. However, what literature is available is consistent with our observation that C_60_ is not inhibitory as it has not been found to negatively affect the growth, gas production or composition of microbial wastewater communities, although the exact microorganisms present in these communities are not specified (Nyberg et al., 2008; Zhao et al., 2018).

Our results contrast with some previous data. It has previously been found that C_60_ at 2.5 mg/L or higher is inhibitory to both *Escherichia coli* and *Bacillus subtilis* when grown both aerobically and anaerobically (Fortner et al., 2005). This concentration is significantly lower than that used in our experiments, and yet we do not observe this reported biotoxicity. However, Fortner et al. conducted their experiments with two species in pure cultures. In a community, however, we hypothesize that the diversity of species allows for some redundancy. It is more likely that even if some species are susceptible to C_60_ toxicity, others are better adapted to withstand it and will therefore become more dominant and able to grow, which may explain why we do not observe any inhibition. Interestingly, Auwerter et al. (2017) found that although lower concentrations of C_60_ did not affect the anaerobic community in their study when these microbes were exposed to higher concentrations, the number of microbes was initially reduced. However, after prolonged exposure, certain species possessing higher resistance to C_60_ were selected for and began to significantly increase in abundance (Auwerter et al., 2017). This is consistent with our proposal that microbial communities possess redundancy that makes them better able to resist fullerene biotoxicity. Certain species in the community used in our study may have been better equipped to use C_60_ and therefore when these species become more dominant through compositional shift, we observe the community as a whole to be growing on C_60_ as a sole carbon source.

We obtained TEM images of the anaerobic community after growth with C_60_ as the sole carbon source. We observed that some of the C_60_ in these cultures and non-biological controls formed flake-like structures in the M9 media, probably due to the salt content causing larger aggregates to form (Lyon et al., 2005) but confirmed with Raman spectroscopy that these still consisted of C_60_. The particle sizes in these images are consistent with the hydrodynamic particle radii we obtained from DLS measurements of the fullerene suspensions. Some of the C_60_ did remain in smaller aggregates that were similar in size and appearance to those observed under TEM by Lyon et al. (2006).

A particularly interesting feature present in our TEM images is the small black spherical clusters on the surface of the bacterial cells consistently visible when the anaerobic community was cultured with C_60_ as the sole carbon source. Similar structures have been previously observed under atomic force microscopy of *E. coli* after incubation with water-soluble fullerene derivatives, where it was proposed that these clusters were evidence of the fullerene derivative penetrating the microbial cells (Deryabin et al., 2014). Although in this case, the derivatives were antibacterial and it seemed this penetration resulted in detrimental electrostatic binding to cellular structures, the similarities between these and our TEM images suggest that membrane penetration of C_60_ clusters might be involved in the mechanism of penetration into the cells of the anaerobic community.

The anaerobic isolate, unlike the community, did not seem to be able to use C_60_ as a sole carbon source, although it was not inhibited as long as glucose was available. There are two possible reasons for this discrepancy. First, the community may be capable of degrading C_60_ only via a collaborative mechanism that relies on the combined biodegradation processes from multiple different metabolically diverse species. Syntrophic degradation of hydrocarbons, including PAHs (polycyclic aromatic hydrocarbons), has been reported previously (Gieg et al., 2014; Harindintwali et al., 2023) and it could be that different organisms can contribute different enzymatic capabilities that together enhance the degradation of fullerenes. Second, it may be that only a select few species present in the community are capable of using C_60_ and that the single species we isolated and cultured independently did not possess this ability. This would be consistent with the suggestion that the complexity of the microbial community results in a certain degree of redundancy that proves to be essential for the degradation of complex organics such as fullerenes.

### 4.2 Fullerene degradation under UV radiation and microbial growth

C_60_ aggregates in water, in the presence of oxygen, degrade readily when exposed to UV radiation, and the degradation products are less aggregated and have increased water stability (Hou et al., 2010; Hou and Jafvert, 2009; Lee et al., 2009; Sanchís et al., 2018). In our studies, the average size of the aggregates was seen to grow in M9 media when exposed to UVC under anoxic conditions for two weeks, although the total amount of carbon incorporated within these aggregates reduced with time. We also observed a distinct color change after irradiation that is consistent with the larger aggregate size. In the absence of oxygen, UV irradiation is known to induce photopolymerization of C_60_ by a 2 + 2 cycloaddition reaction via the triplet excited state (Zhou et al., 1993), however, we saw no evidence for polymerization products in the Raman spectra of the irradiated supernatant or precipitated material. The UVC irradiation of H_2_O is known to produce hydrogen peroxide and radical species such as HO_2_ and OH that can react with the fullerene cage to produce oxidized species, similar to the reaction products found in the presence of oxygen (Ou et al., 2019; Piskarev et al., 2018). These reactions are found to be enhanced for fullerenes in the triplet photoexcited state (Ou et al., 2019). Raman spectra of the irradiated fullerenes show that the cage structure remains intact but indicate the presence of oxidation by the small shift to lower wavenumbers and broadening of peaks (Supplementary Figure 7).

The anaerobic isolate was cultured with the irradiation products from C_60_, both with and without glucose, and we found the resulting cell densities to be statistically comparable to the positive control in both cases. This suggests that not only are the C_60_ UV irradiation products used by the isolate as a sole carbon source, but they also appear to be equally as accessible as glucose. This does seem to be consistent with results from previous studies; it is known that the combination of photodegradation and biodegradation significantly increases the rate of C_60_ mineralization in soils (Peng et al., 2020), and this enhanced degradation of UV-irradiated C_60_ has been observed in soil microcosms (Berry et al., 2016).

### 4.3 The effect of fullerene size on microbial growth and inhibition

The effect of C_70_ on both the community and the isolate deviated from what we observe with C_60_. The community, while able to utilize C_60_, did not seem to utilize C_70_ as a carbon source. If glucose was available, then the community was still able to grow normally with C_70_ at 100 mg/L, but at the higher concentration of 500 mg/L it becomes significantly inhibitory, even with glucose. A significant difference between the two fullerene species is their photoreactivity. Although triplet-mediated singlet oxygen production (the main source of production of reactive oxygen species and resulting toxicity in the presence of oxygen) is not relevant under the anoxic conditions of relevance here, there could still be triplet mediated processes in the complex M9 environment that lead to the observed differences.

Similarly to the results observed with C_60_, we observed differences between the community and the isolate. The isolate was not able to grow with C_70_, with or without glucose. This susceptibility to C_70_ toxicity could be a reflection of a species specific effect which is not seen in the community because of the redundancy provided by the community, in which some species may be better adapted to resist complete inhibition, while the isolate is evidently not. These results again underscore the importance in understanding community-level effects of these compounds on total community function. Results using isolated strains of microorganisms, although important, may not represent how whole communities respond to these compounds in natural environments.

### 4.4 The effect of fullerols on microbial growth and inhibition

The soluble fullerol derivatives of C_60_ and C_70_ exhibit significantly different effects on the anaerobic microbes in this study compared with their underivatised counterparts. While C_60_ seemed biologically accessible to the anaerobic community, C_60_ fullerol is inhibitory under identical growth conditions, even with supplementary glucose, although this inhibition seems to be reduced when cultures were grown in the dark. This trend is also observed with the anaerobic isolate, which is significantly inhibited by C_60_ fullerol in ambient light but not in the dark. When we exposed C_60_ fullerol suspensions to UV radiation under an anoxic atmosphere, they became significantly more inhibitory to both the community and the isolate, but only if the cultures were grown under prolonged ambient light exposure after inoculation. Interestingly, while C_70_ fullerene was inhibitory to both the community and isolate to varying degrees, C_70_ fullerol has no effect on the growth of either, provided additional glucose is available, but the lack of available literature on the effects of C_70_ and its fullerol derivative makes it difficult to form any consensus on the reproducibility of this observation. The difference between light and dark conditions indicates that the photoreactivity is key. Infrared spectra show that after prolonged exposure to light there was a reduction in the number of OH ligands attached to the cage (Supplementary Material, Figure 8). The OH radical is highly oxidizing in nature and the release of this ROS could be the reason for the inhibitory nature of the fullerol species in these experiments.

Several previous studies have found that C_60_ fullerol does not exhibit antibacterial activity despite its photoactivity (Chiang et al., 1995; Li et al., 2008), and is even used by soil microbes as a carbon source (Berry et al., 2017). This is contradictory to our results; however, a major difference is that these previous studies were conducted aerobically and ours was carried out with anaerobic species in an anoxic environment. ROS are generated intracellularly as a product of aerobic metabolism, and therefore aerobic bacteria possess mechanisms to counteract the effects of oxidation and can tolerate low levels of ROS (Nasim and Hamblin, 2017). Anaerobes, on the other hand, can escape oxidative damage by living in anoxic habitats and therefore some may not have mechanisms to mitigate the damage of reactive oxygen (Seixas et al., 2022). It is therefore possible that the aerobic species from previous studies are less susceptible to fullerol biotoxicity than the anaerobes used in our experiments as they are better equipped to deal with the oxidative stress fullerols exert.

C_60_ fullerol produces reactive oxygen species (ROS) when exposed to both UV radiation and visible light (Pickering and Wiesner, 2005). ROS are generated at a much higher rate under UV radiation (Pickering and Wiesner, 2005), which may explain why the greatest biotoxicity we observed was from irradiated C_60_ fullerol which was kept under prolonged visible light exposure during culturing as this condition likely produces the highest ROS concentration. Additionally, ROS tend to be short-lived due to their high reactivity, therefore those produced under UV radiation are likely neutralized quickly within the suspension and therefore do not have a chance to exert oxidative effects on the microbes when incubated in the dark. When under constant visible light exposure, however, this supply of ROS may be continually generated, leading to the greater antibacterial activity we observed in the cultures kept in ambient light. This effect is important from an early Earth perspective as constant UV radiation exposure would presumably increase the biotoxicity of any fullerols in the environment.

If ROS generation is indeed the cause of the biotoxicity we observed when microorganisms are cultured with fullerols, then we might expect to find the same inhibition in the C_60_ suspensions as previous studies claim to have detected ROS production in photosensitized C_60_ suspensions (Kamat et al., 2000). However, Lyon et al. (2008) did not observe any ROS-mediated inhibition to the growth of *E. coli* or *B. subtilis* after exposure to C_60_, and neither did they detect any ROS production in the suspensions (Lyon et al., 2008). After further investigation, these authors propose that C_60_ itself reacts with the dyes used in ROS colorimetric assays, giving false positive results, and that in actuality fullerene suspensions do not produce ROS in any detectable amount (Lyon et al., 2008). Hotze et al. (2008) similarly went on to explain the absence of ROS in UV-irradiated C_60_ suspensions by comparing the density of fullerene aggregates to their fullerol derivatives: more densely aggregated C_60_ is less likely to produce singlet oxygen than the more loosely packed fullerol derivative (Hotze et al., 2008). The less densely-packed fullerols have a larger fullerol-water interface, favoring ROS production, as well as a longer-lived triplet state which leads to a higher generation of singlet oxygen (Hotze et al., 2008).

The microbial accessibility of UV-degraded C_60_ is consistent with the conjecture that microbes on the early Earth could have used extra-terrestrial fullerenes as a carbon source, but the toxicity of their soluble derivatives may counteract this to some extent. Before speculating on the significance of fullerol toxicity on the early Earth, it is important to consider whether or not fullerols would even have been present in this environment. A recent study by Sanchís et al. (2018) concluded that fullerols are not produced from UV exposure in fullerene water suspensions, and so if this is the case, it is possible that fullerols might not have been present on the early Earth in high enough quantities to make them a significant component of the various fullerenes to which early organisms were exposed.

## 5 Conclusion

Our findings indicate that fullerene C_60_ is biologically accessible for utilization as a carbon source by an anaerobic microbial community. We do not observe this when a single species from this community is grown in isolation, which indicates either C_60_ degradation is a collaborative mechanism, or the isolate selected does not possess the individual ability to metabolize fullerenes as well as other species in the community. When C_60_ is exposed to environmental conditions relevant to the early Earth, namely short-wave UV radiation exposure in anoxic media, the degradation products provide an accessible carbon source for the anaerobic isolate, which is more indicative of how fullerenes may have supported early heterotrophic growth. C_60_ fullerol is highly inhibitory to both the community and the isolate, particularly when exposed to UV radiation and kept under ambient light. However, it is possible that fullerols are not produced from fullerene suspensions under environmental conditions in any amount sufficient to inhibit growth, and therefore their biotoxicity may not be as relevant when considering early microbes and their response to extra-terrestrial fullerenes. C_70_ fullerene is not accessible as a carbon source for the anaerobic microorganisms in this study, and at sufficient concentrations can be inhibitory. The difference in the behavior of C_60_ and C_70_ may be attributable to the difference in photophysical properties and chemical reactivity.

Our work shows how fullerenes and their derivatives could potentially have interacted with anaerobic life on a young Earth. Although the presence of a carbon source is assumed to be good for habitability, we show that microbial interactions with these molecules under anoxic conditions are complex, providing a food source or being inhibitory depending on environmental conditions. These results also have implications for the environmental impact of these industrially-produced compounds in anoxic environments on Earth in the present day.

## Data Availability

The data presented in the study are deposited in the Datashare repository, accession: https://datashare.ed.ac.uk/handle/10283/8828.

## References

[B1] Auwerter L. C. C. Ouki S. K. Asaadi M. Shana A. (2017). Effects of nanosized titanium dioxide (TiO_2_) and fullerene (C_60_) on wastewater microorganisms activity. *J. Water Process Eng.* 16 35–40. 10.1016/j.jwpe.2016.12.006

[B2] Avanasi R. Jackson W. A. Sherwin B. Mudge J. F. Anderson T. A. (2014). C_60_ fullerene soil sorption, biodegradation, and plant uptake. *Environ. Sci. Technol.* 48 2792–2797. 10.1021/es405306w 24521447

[B3] Berry T. D. Clavijo A. P. Zhao Y. Jafvert C. T. Turco R. F. Filley T. R. (2016). Soil microbial response to photo-degraded C_60_ fullerenes. *Environ. Pollut.* 211 338–345. 10.1016/j.envpol.2015.12.025 26774781

[B4] Berry T. D. Filley T. R. Clavijo A. P. Bischoff Gray M. Turco R. (2017). Degradation and microbial uptake of C_60_ fullerols in contrasting agricultural soils. *Environ. Sci. Technol.* 51 1387–1394. 10.1021/acs.est.6b04637 28024122

[B5] Botta O. Bada J. L. (2002). Extraterrestrial organic compounds in meteorites. *Surv. Geophys.* 23 411–467. 10.1023/A:1020139302770

[B6] Brant J. A. Labille J. Robichaud C. O. Wiesner M. (2007). Fullerol cluster formation in aqueous solutions: Implications for environmental release. *J. Colloid Interface Sci.* 314 281–288. 10.1016/J.JCIS.2007.05.020 17583721

[B7] Brunet L. Lyon D. Y. Hotze E. M. Alvarez P. J. J. Wiesner M. R. (2009). Comparative photoactivity and antibacterial properties of C_60_ fullerenes and titanium dioxide nanoparticles. *Environ. Sci. Technol.* 43 4355–4360. 10.1021/ES803093T 19603646

[B8] Chae S.-R. Hotze E. M. Wiesner M. R. (2009). Evaluation of the oxidation of organic compounds by aqueous suspensions of photosensitized hydroxylated-C_60_ fullerene aggregates. *Environ. Sci. Technol.* 43 6208–6213. 10.1021/ES901165Q 19746715

[B9] Chae S. R. Hunt D. E. Ikuma K. Yang S. Cho J. Gunsch C. K. (2014). Aging of fullerene C_60_ nanoparticle suspensions in the presence of microbes. *Water Res.* 65 282–289. 10.1016/j.watres.2014.07.038 25150515

[B10] Chiang L. Y. Lu F. J. Lin J. T. (1995). Free radical scavenging activity of water-soluble fullerenols. *J. Chem. Soc. Chem. Commun.* 0 1283–1284. 10.1039/C39950001283

[B11] Chyba C. F. Thomas P. J. Brookshaw L. Sagan C. (1990). Cometary delivery of organic molecules to the early earth. *Science* 249 366–373. 10.1126/science.11538074 11538074

[B12] Cnossen I. Sanz-Forcada J. Favata F. Witasse O. Zegers T. Arnold N. F. (2007). Habitat of early life: Solar X-ray and UV radiation at Earth’s surface 4–3.5 billion years ago. *J. Geophys. Res. Planets* 112:2008. 10.1029/2006JE002784

[B13] Cockell C. S. (1998). Biological effects of high ultraviolet radiation on rarly Earth-a theoretical evaluation. *J. Theor. Biol.* 193 717–729. 10.1006/jtbi.1998.0738 9745762

[B14] Deryabin D. G. Davydova O. K. Yankina Z. Z. Vasilchenko A. S. Miroshnikov S. A. Kornev A. B. (2014). The activity of [60]fullerene derivatives bearing amine and carboxylic solubilizing groups against *Escherichia coli*: A comparative study. *J. Nanomater.* 2014. 10.1155/2014/907435

[B15] Duncan L. K. Jinschek J. R. Vikesland P. J. (2008). C_60_ colloid formation in aqueous systems: Effects of preparation method on size, structure, and surface charge. *Environ. Sci. Technol.* 42 173–178. 10.1021/es071248s 18350893

[B16] Eaton A. D. Franson M. A. H. (2005). *Standard Methods for the Examination of Water and Wastewater*, 21st Edn. Washington, DC: American Public Health Association.

[B17] Ehrenfreund P. Spaans M. Holm N. G. (2011). The evolution of organic matter in space. *Philos. Trans. R. Soc. Math. Phys. Eng. Sci.* 369 538–554. 10.1098/rsta.2010.0231 21220279

[B18] Fang J. Lyon D. Y. Wiesner M. R. Dong J. Alvarez P. J. J. (2007). Effect of a fullerene water suspension on bacterial phospholipids and membrane phase behavior. *Environ. Sci. Technol.* 41 2636–2642. 10.1021/es062181w 17438827

[B19] Fortner J. D. Lyon D. Y. Sayes C. M. Boyd A. M. Falkner J. C. Hotze E. M. (2005). C_60_ in water: Nanocrystal formation and microbial response. *Environ. Sci. Technol.* 39 4307–4316. 10.1021/es048099n 15984814

[B20] Gieg L. M. Fowler S. J. Berdugo-Clavijo C. (2014). Syntrophic biodegradation of hydrocarbon contaminants. *Curr. Opin. Biotech.* 27 21–29. 10.1016/j.copbio.2013.09.002 24863893

[B21] Hancock D. E. Indest K. J. Gust K. A. Kennedy A. J. (2012). Effects of C_60_ on the *Salmonella typhimurium* TA100 transcriptome expression: Insights into C_60_-mediated growth inhibition and mutagenicity. *Environ. Toxicol. Chem.* 31 1438–1444. 10.1002/etc.1848 22511527

[B22] Harindintwali J. D. Xiang L. Wang F. Chang S. X. Zhao Z. Mei Z. (2023). Syntrophy of bacteria and archaea in the anaerobic catabolism of hydrocarbon contaminants. *Crit. Rev. Environ. Sci. Techn.* 53 1331–1357. 10.1080/10643389.2022.2134702

[B23] Hotze E. M. Badireddy A. R. Chellam S. Wiesner M. R. (2009). Mechanisms of bacteriophage inactivation via singlet oxygen generation in UV illuminated fullerol suspensions. *Environ. Sci. Technol.* 43 6639–6645. 10.1021/ES901110M 19764229

[B24] Hotze E. M. Labille J. Alvarez P. Wiesner M. R. (2008). Mechanisms of photochemistry and reactive oxygen production by fullerene suspensions in water. *Environ. Sci. Technol.* 42 4175–4180. 10.1021/ES702172W 18589984

[B25] Hou W. C. Jafvert C. T. (2009). Photochemical transformation of aqueous C clusters in sunlight. *Environ. Sci. Technol.* 43 362–367. 10.1021/es802465z 19238965

[B26] Hou W. C. Kong L. Wepasnick K. A. Zepp R. G. Fairbrother D. H. Jafvert C. T. (2010). Photochemistry of aqueous C_60_ clusters: Wavelength dependency and product characterization. *Environ. Sci. Technol.* 44 8121–8127. 10.1021/es101230q 20939530

[B27] Huang L. Wang M. Dai T. Sperandio F. F. Huang Y. Y. Xuan Y. (2014). Antimicrobial photodynamic therapy with decacationic monoadducts and bisadducts of [70]fullerene: In vitro and in vivo studies. *Nanomed.* 9 253–266. 10.2217/NNM.13.22/SUPPL_FILE/SUPPL_INFORMATION.DOCX PMC3859801 23738632

[B28] Huang L. Wang M. Sharma S. Sperandio F. Maragani S. Nayka S. (2013). Decacationic [70]Fullerene approach for efficient photokilling of infectious bacteria and cancer cells. *ECS Trans.* 45 65–73. 10.1149/04520.0065ECST/XML PMC3880553 24396566

[B29] Hwang Y. S. Li Q. (2010). Characterizing photochemical transformation of aqueous nC_60_ under environmentally relevant conditions. *Environ. Sci. Technol.* 44 3008–3013. 10.1021/es903713j 20337472

[B30] Kamat J. P. Devasagayam T. P. A. Priyadarsini K. I. Mohan H. (2000). Reactive oxygen species mediated membrane damage induced by fullerene derivatives and its possible biological implications. *Toxicology* 155 55–61. 10.1016/S0300-483X(00)00277-8 11154797

[B31] Kokubo K. Shirakawa S. Kobayashi N. Aoshima H. Oshima T. (2011). Facile and scalable synthesis of a highly hydroxylated water-soluble fullerenol as a single nanoparticle. *Nano Res.* 4 204–215. 10.1007/s12274-010-0071-z

[B32] Kroto H. (1988). Space, stars, C_60_, and soot. *Science* 242 1139–1145. 10.1126/science.242.4882.1139 17799730

[B33] Kyzyma O. A. Avdeev M. V. Bolshakova O. I. Melentev P. Sarantseva S. V. Ivankov O. I. (2019). State of aggregation and toxicity of aqueous fullerene solutions. *Appl. Surf. Sci.* 483 69–75. 10.1016/j.apsusc.2019.03.167

[B34] Lee J. Cho M. Fortner J. D. Hughes J. B. Kim J. H. (2009). Transformation of aggregated C_60_ in the aqueous phase by UV irradiation. *Environ. Sci. Technol.* 43 4878–4883. 10.1021/es8035972 19673279

[B35] Leroy M. Jassey V. E. Silvestre J. Barret M. Flahaut E. Larue C. (2024). Carbon nanotubes alter agrosystem multifunctionality. *Enviro. Sci.* 11 4126–4137. 10.1039/D4EN00195H

[B36] Li Q. Mahendra S. Lyon D. Y. Brunet L. Liga M. V. Li D. (2008). Antimicrobial nanomaterials for water disinfection and microbial control: Potential applications and implications. *Water Res.* 42 4591–4602. 10.1016/J.WATRES.2008.08.015 18804836

[B37] Liu W. Wang Z. Chai G. Deng W. (2023). Effect of carbon nanomaterials on functional diversity and structure of soil microbial community under single and repeated exposures. *Environ. Sci. Poll. Res.* 30 115896–115906. 10.1007/s11356-023-30653-y 37897582

[B38] Lyon D. Y. Adams L. K. Falkner J. C. Alvarez P. J. J. (2006). Antibacterial activity of fullerene water suspensions: Effects of preparation method and particle size. *Environ. Sci. Technol.* 40 4360–4366. 10.1021/es0603655 16903271

[B39] Lyon D. Y. Brunet L. Hinkal G. W. Wiesner M. R. Alvarez P. J. J. (2008). Antibacterial activity of fullerene water suspensions (nC_60_) is not due to ROS-mediated damage. *Nano Lett.* 8 1539–1543. 10.1021/NL0726398 18410152

[B40] Lyon D. Y. Fortner J. D. Sayes C. M. Colvin V. L. Hughes J. B. (2005). Bacterial cell association and antimicrobial activity of a C_60_ water suspension. *Environ. Toxicol. Chem.* 24 2757–2762. 10.1897/04-649R.1 16398110

[B41] Ma J. Guo R. Tan X. (2020). Aqueous photochemistry of fullerol revisited: Energy transfer vs. electron transfer processes probed by Rhodamine B degradation. *J. Photochem. Photobiol. Chem.* 397:112600. 10.1016/J.JPHOTOCHEM.2020.112600

[B42] Maier J. P. Campbell E. K. (2017). Fullerenes in space. *Angew. Chem. Int. Ed.* 56 4920–4929. 10.1002/anie.201612117 28070989

[B43] Nasim K. Hamblin M. R. (2017). Can microbial cells develop resistance to oxidative stress in antimicrobial photodynamic inactivation? Drug Resist. Updat. Rev. Comment. *Antimicrob. Anticancer Chemother.* 31:31. 10.1016/j.drup.2017.07.003 28867242 PMC5673603

[B44] Navarro D. A. Kookana R. S. McLaughlin M. J. Kirby J. K. (2015). Fullerol as a potential pathway for mineralization of fullerene nanoparticles in biosolid-amended soils. *Environ. Sci. Technol. Lett.* 3 7–12. 10.1021/ACS.ESTLETT.5B00292

[B45] Nyberg L. Turco R. F. Nies L. (2008). Assessing the impact of nanomaterials on anaerobic microbial communities. *Environ. Sci. Technol.* 42 1938–1943. 10.1021/es072018g 18409617

[B46] Ou Y. Wu J. Meyer J. R. Foston M. Fortner J. D. Li W. (2019). Photoenhanced oxidation of nC_60_ in water: Exploring H_2_O_2_ and hydroxyl radical based reactions. *Chem. Eng. J.* 360 665–672. 10.1016/j.cej.2018.12.035

[B47] Ouyang K. Dai K. Walker S. L. Huang Q. Yin X. Cai P. (2016). Efficient photocatalytic disinfection of *Escherichia coli* O157:H7 using C_70_-TiO_2_ hybrid under visible light irradiation. *Nat. Publ. Group* 6:25702. 10.1038/srep25702 27161821 PMC4861983

[B48] Pasek M. Lauretta D. (2008). Extraterrestrial flux of potentially prebiotic C, N, and P to the early earth. *Orig. Life Evol. Biospheres* 38 5–21. 10.1007/s11084-007-9110-5 17846915

[B49] Peng Z. Liu X. Zhang W. Zeng Z. Liu Z. Zhang C. (2020). Advances in the application, toxicity and degradation of carbon nanomaterials in environment: A review. *Environ. Int.* 134:105298. 10.1016/j.envint.2019.105298 31765863

[B50] Pickering K. D. Wiesner M. R. (2005). Fullerol-sensitized production of reactive oxygen species in aqueous solution. *Environ. Sci. Technol.* 39 1359–1365. 10.1021/ES048940X 15787378

[B51] Piskarev I. M. Astaf’eva K. A. Ivanova I. P. (2018). The UV-C radiation mechanism to form HO_2_ radicals from water. *J. Phys. Conf. Ser.* 1094:012024. 10.1088/1742-6596/1094/1/012024

[B52] Riding M. J. Martin F. L. Trevisan J. Llabjani V. Patel I. I. Jones K. C. (2012). Concentration-dependent effects of carbon nanoparticles in Gram-negative bacteria determined by infrared spectroscopy with multivariate analysis. *Environ. Pollut.* 163 226–234. 10.1016/j.envpol.2011.12.027 22265761

[B53] Ruoff R. S. Tse D. S. Malhotra R. Lorents D. C. (1993). Solubility of C_60_ in a variety of solvents. *J. Phys. Chem.* 97 3379–3383. 10.1021/j100115a049

[B54] Sabbah H. Carlos M. Jenniskens P. Shaddad M. H. Duprat J. Goodrich C. A. (2022). Detection of cosmic fullerenes in the Almahata Sitta Meteorite: Are they an interstellar heritage? *Astrophys. J.* 931:91. 10.3847/1538-4357/ac69dd

[B55] Sanchís J. Aminot Y. Abad E. Jha A. N. Readman J. W. Farré M. (2018). Transformation of C_60_ fullerene aggregates suspended and weathered under realistic environmental conditions. *Carbon* 128 54–62. 10.1016/j.carbon.2017.11.060

[B56] Schönheit P. Buckel W. Martin W. F. (2016). On the origin of heterotrophy. *Trends Microbiol.* 24 12–25. 10.1016/j.tim.2015.10.003 26578093

[B57] Seixas A. F. Quendera A. P. Sousa J. P. Silva A. F. Q. Arraiano C. M. Andrade J. M. (2022). Bacterial response to oxidative stress and RNA oxidation. *Front. Genet.* 12:821535. 10.3389/fgene.2021.821535 35082839 PMC8784731

[B58] Semenov K. N. Charykov N. A. Postnov V. N. Sharoyko V. V. Vorotyntsev I. V. Galagudza M. M. (2016). Fullerenols: Physicochemical properties and applications. *Prog. Solid State Chem.* 44 59–74. 10.1016/j.progsolidstchem.2016.04.002

[B59] Taylor R. Parsons J. P. Avent A. G. Rannard S. P. Dennis T. J. Hare J. P. (1991). Degradation of C_60_ by light. *Nature* 351:277. 10.1038/351277A02034272

[B60] Tsao N. Luh T. Y. Chou C. K. Chang T. Y. Wu J. J. Liu C. C. (2002). In vitro action of carboxyfullerene. *J. Antimicrob. Chemother.* 49 641–649. 10.1093/jac/49.4.641 11909838

[B61] Waajen A. C. Prescott R. Cockell C. S. (2022). Meteorites as food source on early Earth: Growth, selection, and inhibition of a microbial community on a carbonaceous chondrite. *Astrobiology* 22 495–508. 10.1089/ast.2021.0089 35319269

[B62] Wang J. Ma Q. Zhang Z. Diko C. Qu Y. (2020). Biogenic Fenton-like reaction involvement in anaerobic degradation of C_60_ by *Labrys* sp. WJW. *Environ. Pollut.* 272:115300. 10.1016/j.envpol.2020.115300 33279268

[B63] Zhao L. Ji Y. Sun P. Li R. Xiang F. Wang H. (2018). Effects of individual and complex ciprofloxacin, fullerene C_60_, and ZnO nanoparticles on sludge digestion: Methane production, metabolism, and microbial community. *Bioresour. Technol.* 267 46–53. 10.1016/j.biortech.2018.07.024 30014997

[B64] Zhou P. Dong Z.-H. Rao A. M. Eklund P. C. (1993). Reaction mechanism for the photopolymerization of solid fullerene C_60_. *Chem. Phys. Lett.* 211 337–340. 10.1016/0009-2614(93)87069-F

[B65] Zuo Y. Zeng W. Huang J. (2024). Effects of exposure to carbon nanomaterials on soil microbial communities: A global meta-analysis. *Land Degrad. Develop.* 35 238–248. 10.1002/ldr.4912

[B66] Zygouri P. Spyrou K. Mitsari E. Barrio M. Macovez R. Patila M. (2020). A facile approach to hydrophilic oxidized fullerenes and their derivatives as cytotoxic agents and supports for nanobiocatalytic systems. *Sci. Rep.* 10:8244. 10.1038/s41598-020-65117-7 32427871 PMC7237490

