# Supplementary material for: Harnessing cosmic carbon: anaerobic microbial responses to fullerenes under early Earth conditions
Source: Front Microbiol. 2025 Aug 4;16:1511842. doi: 10.3389/fmicb.2025.1511842 (PMC12360298; doi:10.3389/fmicb.2025.1511842)
Supplement: Supplementary file 1 [file Supplementary_file_1.docx]

**Supplementary Materials**

**Harnessing Cosmic Carbon: Anaerobic Microbial Responses to Fullerenes Under Early Earth Conditions**

Elle Bethune^1*^, Andrey Gromov^2^, Eleanor E.B. Campbell^2,3^, Charles S. Cockell^1^

^1^UK Centre for Astrobiology, School of Physics and Astronomy, University of Edinburgh, Edinburgh, Scotland

^2^EaStCHEM and School of Chemistry, University of Edinburgh, Edinburgh, Scotland

^3^Dept. of Physics, Ewha Womans University, Seoul 03760, Republic of Korea

**Fullerol Characterisation**

The identification of the fullerol materials is based on the combination of FTIR, ^13^C NMR and TGA results shown below.

*FTIR Spectra*

FTIT spectra of dried powder samples of the synthesised fullerol species were obtained using a Smiths Illuminat-IR FTIR microscope equipped with an ATR diamond-coated objective and are shown in Fig. 1. The samples show the expected characteristic peaks. The broad signal at around 3400 cm^-1^ is associated with the O-H stretch. The C=C, C-O-H and C-O frequencies are found at 1625/1627 cm^-1^, 1371/1363 cm^-1^ and 1082/1074 cm^-1^ for the C_60_ (Fig. 1(a)) and C_70_ fullerols (Fig. 1(b)), respectively. The C_60_ fullerol spectrum shows a close resemblance to the spectrum for C_60_(OH)_44_.8H_2_O shown by Kokubo et al. (Kokubo et al., 2011). The small peak at ca. 1720 cm^-1^ is indicative of some carbonyl presence.


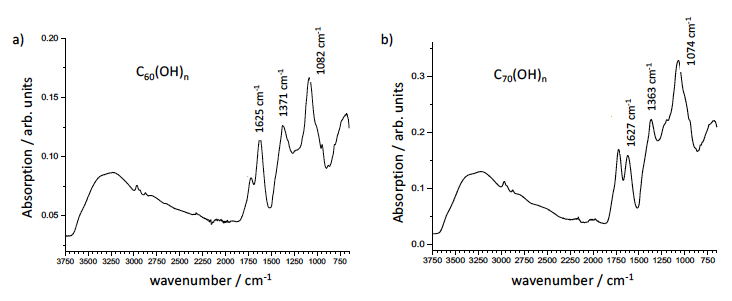


Figure 1: FTIR spectra of synthesised fullerols of (a) C_60_(OH)*_n_* and (b) C_70_(OH)*_n_*.

*^13^C NMR*

The NMR spectra were taken using a Bruker Advance 300 solid-state NMR spectrometer, equipped with a 4 mm WB MAS broadband probe, at a spin rate of 10 kHz. Each spectrum consisted of 1024 scans with a scan time of 3 seconds and are shown in Fig. 2. The integrated sp^2^ and sp^3^ intensities are provided in Table 1.


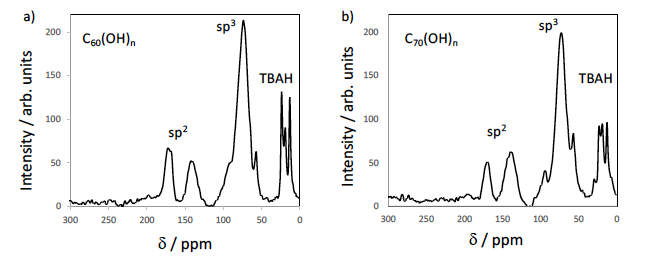


Figure 2: Solid state ^13^C NMR spectra (a) C_60_(OH)*_n_* (b) C_70_(OH)*_n_*. Peaks labelled TBAH are due to trace impurities of the phase transfer catalyst used in synthesis.

Table 1. ^13^C NMR Integrated Peak Intensities (arb. units)

| **C_60_(OH)*n*** | | | | |
| --- | --- | --- | --- | --- |
| ppm | Height | Width | Area | Type |
| 172 | 65.8 | 8.62$\times$10^2^ | 2.70$\times$10^4^ | sp^2^ |
| 141 | 55.8 | 1.18$\times$10^3^ | 3.25$\times$10^4^ | sp^2^ |
| 74 | 225 | 1.33$\times$10^3^ | 1.75$\times$10^5^ | sp^3^ |
| 57 | 32.7 | 2.36$\times$10^2^ | 4.40$\times$10^3^ | sp^3^ |
|  |  |  |  |  |
| **C_70_(OH)*n*** | | | | |
| 171 | 49.2 | 8.55$\times$10^2^ | 2.02$\times$10^4^ | sp^2^ |
| 140 | 69.6 | 1.43$\times$10^3^ | 4.95$\times$10^4^ | sp^2^ |
| 95 | 28.0 | 5.99$\times$10^2^ | 9.27$\times$10^3^ | sp^3^ |
| 73 | 212 | 1.27$\times$10^3^ | 1.49$\times$10^5^ | sp^3^ |
| 57 | 51.6 | 3.81$\times$10^2^ | 1.22$\times$10^4^ | sp^3^ |

Although the relative sp^2^ to sp^3^ peak heights can only give an estimate of the degree of hydroxylation, the observed ratios are indicative of a high degree of hydroxylation in agreement with the FTIR results. The sp^2^/sp^3^ ratios are 0.33 for C_60_(OH)*_n_* and 0.41 for C_70_(OH)*_n_* giving *n* = 45 and *n* =50, respectively. The double sp^2^ peaks may again be indicative of the presence of carbonyl moieties.

*Thermogravimetric Analysis*

Thermogravimetric analysis was carried out using a NETZSCH STA 449F1 instrument under nitrogen atmosphere. The results are shown in Fig. 3.


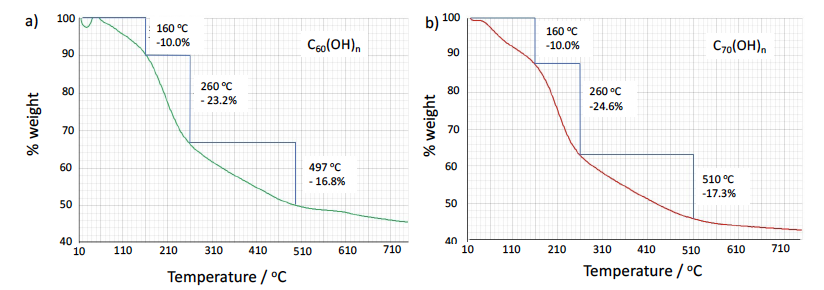


Figure 3: Thermogravimetric analysis (TGA) plots of (a) C_60_(OH)*_n_* (b) C_70_(OH)*_n_*.

The first drop in %weight is attributed to the loss of H_2_O (Chiang et al 1993) . The next two steps are attributed to OH loss (Stankovic et al. 2019). Analysis of the curves provides the brutto chemical formulae of C_60_(OH)_44_(6-8)H_2_O and C_70_(OH)_50_(14-16)H_2_O. The results for the C_60_ fullerol are consistent with (Kokubo et al 2011). The TGA analysis is consistent with the ^13^C NMR and FTIR results.

**Growth of anaerobic community on C_60_**


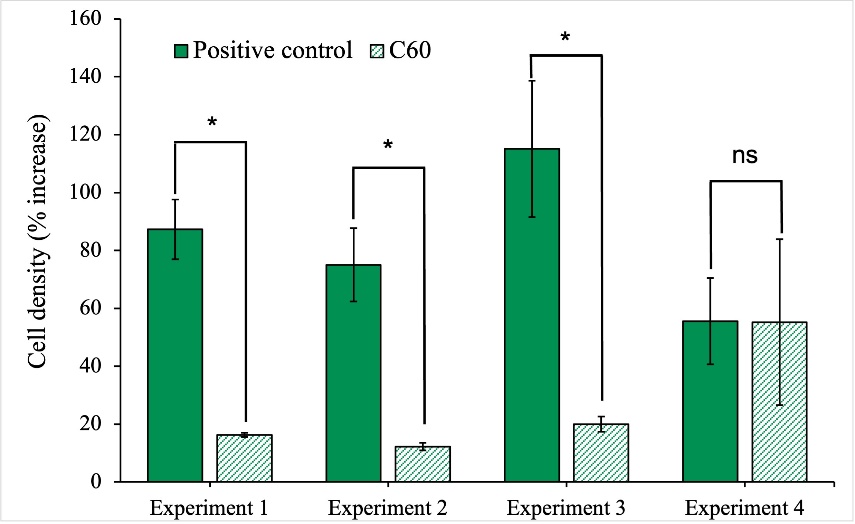


Figure 4: Test of reproducibility of microbial community growth on C_60_. Summary of results from four experiments where the anaerobic community was grown for 28 days with C_60_ (100 mg/L) as a sole carbon source compared to the positive control from each experiment. The significance of the difference between the C_60_ condition and positive control for each experiment is represented by an asterisk (*) where p<0.05 and ‘ns’ where p>0.05.

**Raman Spectroscopy**

*Particles observed in TEM*

A Raman spectrum measured for the carbon deposit identified in the TEM images (main text, Fig. 3) is shown in Fig. 5 and compared with that of pristine C_60_. The spectra were obtained with a Renishaw InVia Raman microscope with a laser wavelength of 785 nm. The most prominent characteristic vibrational modes of C_60_ are clearly seen. In particular the strong A_g_(2) radial breathing mode is a clear indication that the C_60_ cage is intact. The H_g_ modes appear to be lower in intensity, this is most clearly seen for the relatively intense H_g_(1) mode, the others are at the level of the noise. The low frequency modes are also slightly shifted by 2 cm^-1^ to lower wavenumbers compared to the pristine C_60_. Such a small shift in combination with a reduction of the number of observed H_g_ modes was observed previously for oxidised fullerene powder (Zygouri et al 2020) and may be indicating surface oxidation of the particles.


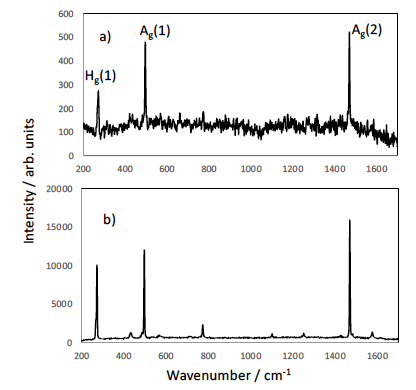


Figure 5: (a) Raman spectrum taken directly from TEM slide containing bacteria and C_60_ (Fig. 3 in main text). (b) Pristine C_60_.

**Microbial community composition**


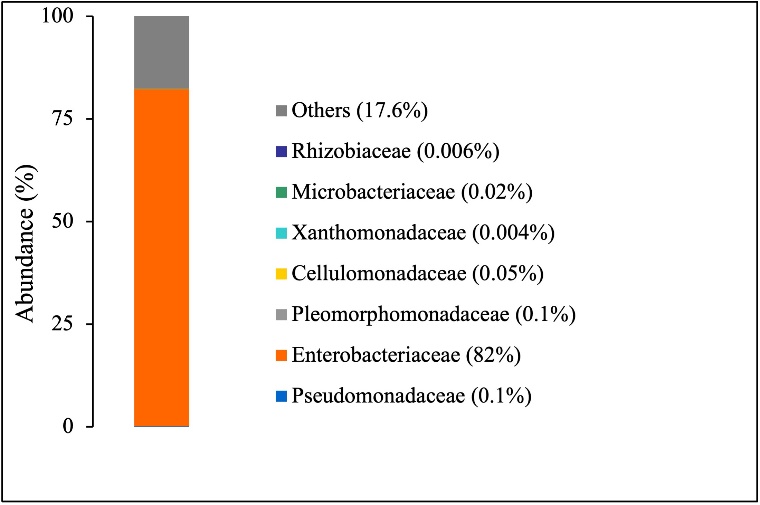


Figure 6: Microbial community composition after 28 days incubation with C_60_. The bar graph and legend show the abundance (as percentages) of each taxon present at the family level. Taxonomic classifications are derived from the Qiime2 database.

**Raman comparison, before and after UV irradiation**

The Raman spectra (785 nm) obtained from C_70_ dispersions in M9 before and after exposure to UV irradiation are shown in Fig. 7. The spectra are very similar with only a broadening and slight shift (1-5 cm^-1^) of peaks to lower wavenumbers in the spectrum obtained after 2 weeks of UVC irradiation.


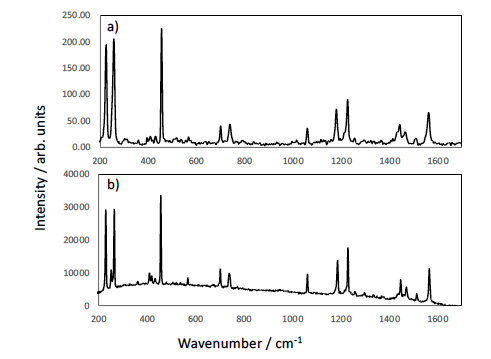


Figure 7: Raman spectra of C_70_ (a) after and (b) before 2 weeks of UVC irradiation.

**FTIR of C_70_(OH)_50_ before and after exposure to ambient light**

The IR spectrum of a C_70_(OH)_50_ dispersion is shown in Fig 8(a), similar to Fig 1(b). The spectrum obtained after exposing the solution to 2 weeks of UVC radiation is shown in Fig 8(b). There is a very large difference in the two spectra with a much smaller signal corresponding to O-H vibrations in the irradiated sample. The results show that the fullerol decays under UV light exposure, losing OH ligands. For comparison, Fig. 8(c) shows the IR spectrum of synthesised C_70_(OH)_18_ which bears a closer resemblance to the light-exposed solution that the starting material, providing additional evidence for the loss of OH ligands.


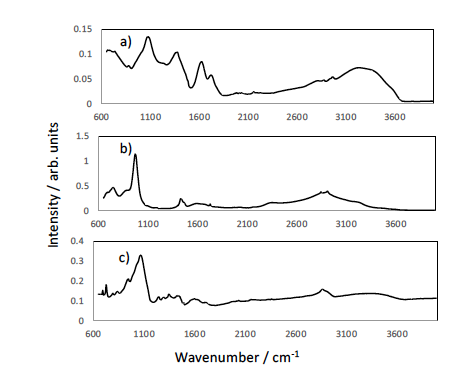


Figure 8: FTIR spectra of C_70_ fullerol before and after 2 week UVC exposure (a) fresh solution, C_70_(OH)_50_ (b) solution after UV exposure (c) spectrum of C_70_(OH)_18_ for comparison.

**References**

Chiang, L.Y., Upasani, R. B., Swirczewski, J. W., Soled, S., 1993. Evidence of hemiketals incorporated in the structure of fullerols derived from aqueous acid chemistry. J. Am. Chem. Soc.*,* 115, 5453-5457. https://pubs.acs.org/doi/10.1021/ja00066a014

Kokubo, K., Shirakawa, S., Kobayashi, N., Aoshima, H., Oshima, T., 2011. Facile and scalable synthesis of a highly hydroxylated water-soluble fullerenol as a single nanoparticle. Nano Res. 4, 204–215. https://doi.org/10.1007/s12274-010-0071-z

Stankovic, B., Jovanovic, J., Adnadjevic, B., 2019. Application of logistic function to describe kinetics of non-isothermal dehydroxylation of fullerol Journal of Thermal Analysis and Calorimetry, 138, 2295-2303.

https://doi.org/10.1007/s10973-019-08222-8

# Zygouri, P., Spyrou, K., Mitsari, E., Barrio, M., Macovez, R., Patila, M., Stamatis, H., Verginadis, I.I., Velalolpoulou, A. P., Evangelou, A.M., Sideratou, Z., Gournis, D., Rudolf, P., 2020. A facile approach to hydrophilic oxidized fullerenes and their derivatives as cytotoxic agents and supports for nanobiocatalytic systems, Sci. Reports 10:8244, https://doi.org/10.1038/s41598-020-65117-7.
